# Psychological outcomes of primary-school physical education interventions: a systematic review and multilevel meta-analysis

**DOI:** 10.3389/fpsyg.2026.1962805

**Published:** 2026-10-07

**Authors:** Lebin Li, Yao Huang, Di Zhang, Jiaqing Meng

**Affiliations:** 1 School of Teacher Education, Southwest University, Chongqing, China; 2 School of Physical Education, Southwest University, Chongqing, China; 3 School of Physical Education, Shanxi Normal University, Taiyuan, China; 4 School of Art, Wuhan Sports University, Wuhan, China

**Keywords:** basic psychological needs, emotion regulation, motivation, multilevel meta-analysis, physical education, self-control, self-efficacy, social–emotional competence

## Abstract

**Introduction:**

Physical education (PE) offers opportunities for psychological learning, but evidence of benefit depends on the outcome, design, and instructional context.

**Methods:**

This systematic review and multilevel meta-analysis included 74 reports from 70 trial families of primary-school PE interventions. Four databases covered English-language reports published from January 2000 through 31 May 2026. Randomized and nonrandomized controlled studies were distinguished from uncontrolled pre -post and short-term within-child designs. Multilevel models addressed dependent effects, with trial-family CR2 inference.

**Results:**

Source-informed controlled analyses contained five core social-emotional effects from three families (g = 0.947, 95% CI [−0.774, 2.667]; Holm-adjusted *p* = 0.424) and 13 adjacent psychological effects from five families (g = 0.215, 95% CI [−0.386, 0.817]; Holm-adjusted *p* = 0.424). Model-based prediction intervals were [−2.387, 4.280] and [−1.368, 1.799], respectively. The broader controlled analysis, requiring additional variance or clustering assumptions, included 126 effects from 28 families and yielded g = 0.381, 95% CI [0.135, 0.627].

**Discussion:**

The broader and combined source-informed estimates were similar (g = 0.381 and 0.421, respectively), although the latter was less precise; because the analyses overlap, they do not provide independent confirmation of benefit. The primary estimates remain too uncertain to define a dependable average benefit, rather than demonstrating that benefits are absent. Positive broader evidence warrants attention alongside substantial heterogeneity, source-reporting limitations, and an inconclusive exploratory small-study diagnostic. Future trials should align psychological targets, instructional content, implementation measures, and validated outcomes, and report allocation-aware uncertainty.

## 1 Introduction

### 1.1 Psychological significance of social–emotional outcomes in physical education

Children’s capacity to understand and regulate emotion, inhibit impulsive responses, cooperate with peers, sustain purposeful effort, and maintain supportive relationships is consequential for participation in both learning and everyday social life. Broad school-based social–emotional learning programs have shown benefits across social–emotional, behavioral, and academic outcomes, including at follow-up. These findings do not establish that ordinary PE lessons or every PE intervention produces the same pattern of change (Durlak et al., 2011; Taylor et al., 2017). A PE-specific synthesis therefore needs to separate the psychological outcome being claimed from the curricular setting in which it is measured.

PE is relevant to sport psychology because it is often children’s first sustained, institutionally organized encounter with movement, performance comparison, competition, cooperation, feedback, success, and failure. It can offer recurring opportunities to act under social evaluation and to coordinate behavior with peers. School PE has long been discussed as a setting with potential physical, social, and educational benefits (Bailey, 2006), yet potential is not evidence of an automatic effect. The psychological meaning of a lesson depends on how tasks, roles, feedback, peer interaction, and reflection are arranged, as well as on how children interpret those experiences.

### 1.2 Psychological domains and non-automatic pathways

The present review distinguishes core social–emotional competencies from adjacent psychological outcomes. Systemic social–emotional learning is a broad educational framework for developing and practicing social, emotional, and academic competencies within aligned school conditions. By contrast, ability-based emotional intelligence models focus more narrowly on appraising, expressing, regulating, and using emotion (Salovey and Mayer, 1990; Mahoney et al., 2021). Emotion regulation refers to processes through which people influence which emotions they have, when they have them, and how those emotions are experienced or expressed (Gross, 2015). Self-control is related but not identical: it concerns goal-aligned action when immediate impulses compete and may begin with managing situations before effortful inhibition is required (Duckworth and Steinberg, 2015; Duckworth et al., 2016). Empathy, prosocial competence, and relationship skills concern understanding and responding to other people, not merely feeling positive during activity. These constructs form the core layer because they directly represent intrapersonal or interpersonal competencies.

The adjacent category includes motivational regulation, perceived competence, self-concept, self-efficacy, basic psychological needs, and wellbeing. These constructs are related but not interchangeable. Self-efficacy concerns capability beliefs for specified actions, whereas competence perceptions and confidence depend on the content and framing of their measures (Bandura, 1977; Usher and Pajares, 2008). Autonomy, competence, and relatedness also have distinct roles in self-determination theory (Deci and Ryan, 2000; Ryan and Deci, 2020; Vansteenkiste et al., 2020). Our core/adjacent distinction is an organizing framework for this review, not a validated division of social–emotional functioning into mutually exclusive entities. CASEL includes self-efficacy within self-awareness, illustrating why plausible frameworks can place the same outcome differently (CASEL, 2026).

Movement exposure alone does not specify how change would occur. A cooperative task may create interdependence, but it becomes a plausible learning pathway only when participation is accountable, peers have meaningful roles, and interaction is facilitated. Responsibility-based teaching can make behavioral expectations and transfer goals explicit, although its label alone does not verify delivery. For reflection to connect an emotionally charged activity with awareness, perspective taking, conflict management, or future action, lessons must provide prompts and time. Model-based PE reviews have connected Teaching Personal and Social Responsibility, cooperative learning, and outdoor or adventure education with some social–emotional outcomes, while also showing incomplete construct coverage and uneven implementation information (Dyson et al., 2021).

Teacher support provides a complementary, psychologically grounded pathway. Choices can support autonomy when they are meaningful rather than cosmetic; feedback can support competence when it is informative and appropriately challenging; and an inclusive climate can support relatedness when belonging is not conditional on performance (Deci and Ryan, 2000; Vasconcellos et al., 2020). These features may influence motivation, enjoyment, perceived competence, self-efficacy, or wellbeing. They cannot, however, be assumed to produce empathy or emotion-regulation skills unless the intervention creates and assesses opportunities for those competencies. Reviews of affective learning in PE similarly emphasize planned outcomes, alignment among aims, teaching, and assessment, and evidence of implementation fidelity (Teraoka et al., 2021).

### 1.3 Review gap, objectives, and research questions

Existing syntheses are encouraging but not uniform. A broad review of children’s physical-activity interventions reported a small-to-moderate social–emotional association alongside substantial heterogeneity (Moon et al., 2024). A review of combined physical-activity and social–emotional programs for adolescents identified only a small set of programs and described mixed, generally modest patterns (Jackson et al., 2024). PE-specific reviews have used broader emotional-intelligence labels, examined model-based pedagogies, or focused on affective learning, but they also identify construct ambiguity, partial coverage, and limited implementation detail (Dyson et al., 2021; Rico-González, 2023; Teraoka et al., 2021). Differences in populations, intervention content, and outcome definitions limit the conclusions that can be drawn specifically for primary-school PE.

The DE-PASS reviews further distinguish intervention targets from behavioral outcomes and organize evidence by setting. Khudair et al. (2025) examined determinants alongside device-measured physical activity and sedentary behavior, whereas Ling et al. (2024) considered self-reported behavior. Both show why a named psychological target or theoretical framework cannot substitute for evidence that the target changed in a particular context. Their broader physical-activity scope complements, but does not directly answer, the present question about curricular PE and social–emotional outcomes.

The evidence base also presents unit-of-analysis and report-identity problems. One trial can generate several articles, outcomes, arms, and endpoints. Shared controls and repeated outcomes create statistical dependence, whereas controlled, uncontrolled pre-post, and acute within-subject designs estimate different contrasts (Assink and Wibbelink, 2016; Hedges et al., 2010). Counting effect rows as independent studies exaggerates the information base, while indiscriminate averaging can obscure psychologically distinct outcomes. A synthesis must therefore keep report, trial-family, and effect-row identities separate and align the model with the inferential target.

Using participants, interventions, comparators, outcomes, and study designs (PICOS), this review asked four questions. First, what is the average controlled association between primary-school PE interventions and core social–emotional competencies? Second, what is the average controlled association with adjacent psychological outcomes? Third, do the two outcome layers differ when compared directly? Fourth, how robust are the conclusions to working dependence assumptions, cluster-design assumptions, design tier, outcome domain, and influential trial families? The review was designed to estimate these associations without assuming that exposure to the PE setting explains how change occurs.

## 2 Methods

### 2.1 Protocol and reporting framework

This review is reported in accordance with PRISMA 2020 (Page et al., 2021). It was not prospectively registered. The authors recall planning the research questions, eligibility criteria, search methods and analytical approach during the development of the review. However, the contemporaneous written protocol or research-design documents could not be recovered, so their original content and timing relative to the initial search, screening and data extraction cannot be verified. Later analysis rules are not presented as an original protocol. Outcome definitions, eligibility decisions, and analytical requirements evolved during the review; documenting them before a particular model fit does not establish that they preceded access to the evidence. The revision retained the submitted dataset as a comparator and recorded source-supported corrections and limited additional analyses. These revision analyses are exploratory. The Supplementary Data workbook distinguishes the submitted comparisons from active revised results, and its Version_Crosswalk and Submitted_Model_Changes sheets record amendment reasons. Supplementary section S12 explains changes from the submitted synthesis.

### 2.2 Eligibility criteria and PICOS

Participants were children in primary or elementary school. Reports with wider age ranges were eligible only when primary-school data were separately extractable or the sampled educational level clearly matched the review scope. Interventions had to be delivered in curricular PE, embedded in PE, or sufficiently isolated from a broader school program to identify the PE component. General physical-activity programs without an identifiable PE context were not treated as equivalent. Eligible publications were English-language, peer-reviewed journal articles or formally published early-access articles issued between January 2000 and 31 May 2026. Early-access and version-of-record items were linked as the same report when applicable; withdrawn or superseded versions were not counted as current evidence.

Eligible comparators included usual PE, an alternative PE curriculum, wait-list or minimal provision, and, for separately described designs, a participant’s own preintervention or short-term comparison condition. Randomized, cluster-randomized, nonrandomized controlled, uncontrolled pre–post, short-term between-group, and within-child designs could enter the systematic review. Sustained controlled contrasts formed the main quantitative evidence. Uncontrolled change scores were analyzed separately. Short-term between-group and within-child contrasts were not combined because their estimands differed and only two between-group families and one within-child family were available.

Eligible outcomes represented at least one core or adjacent psychological domain. Core outcomes were direct social–emotional or emotional-intelligence composites, emotional awareness or expression, emotion regulation or self-management, self-control or impulse control, empathy or prosocial competence, and relationship or social competence. Adjacent outcomes were motivation or enjoyment, autonomy, competence or relatedness, self-efficacy, self-concept or self-esteem, and wellbeing or emotional or behavioral adjustment. Purely cognitive, academic, motor, fitness, physiological, or clinical outcomes without a target psychological construct were outside the outcome scope. Eligibility for the systematic review did not depend on whether a standardized effect could be calculated: reports with relevant evidence remained in the narrative synthesis when their results could not be converted to Hedges’ g without unsupported assumptions.

### 2.3 Information sources and search strategy

Web of Science Core Collection, ERIC, APA PsycINFO via ProQuest, and PubMed were searched for reports published from January 2000 through 31 May 2026. The archived Web of Science export was dated 28 May 2026, and the PsycINFO export was dated 29 May 2026. The exact ERIC execution date was not recoverable from the archived project. The preserved coverage and executable strategy are therefore reported without assigning an unverifiable date. Database-specific strategies, limits, and result counts appear in Supplementary Table S2. PubMed was searched without a language filter; reports not published in English were excluded during eligibility assessment under the review’s language criterion.

The historical PubMed export contained 416 PMIDs, but its executable query and reliable run date were not recoverable. To restore reproducibility, two documented candidate queries were executed through NCBI E-utilities on 30 July 2026 with the publication-date cutoff fixed at 31 May 2026. The selected high-sensitivity query returned 513 records and retrieved all 11 prespecified sentinel reports. It shared 287 PMIDs with the historical export; 226 records were unique to the rerun and 129 historical records were not returned, giving a 642-record union. The exact selected query, response metadata, overlap analysis, and incremental decisions are supplied in the Supplementary materials.

### 2.4 Study selection and report linkage

Records were deduplicated and linked at the report level using DOI, PMID, formal article number, normalized title with author and year, and documented version relationships. Screening outcomes were recorded separately for title–abstract assessment, report retrieval, full-text assessment, and inclusion. Eligibility for the systematic review was distinguished from eligibility for meta-analysis, allowing an included report to remain represented when it did not provide a compatible controlled effect. Exact duplicates, errata, withdrawn versions, and companion publications were resolved before the PRISMA counts were finalized.

One author conducted the primary title–abstract screening, full-text assessment, data extraction, effect coding, and MMAT appraisal. As documented for the submitted version, all four authors subsequently reviewed the source-linked decisions and analysis materials. This process did not constitute fully independent duplicate review, and no inter-rater agreement statistic was calculated. Revision-stage checking procedures are described in Supplementary section S10. Source checks and unresolved limitations are documented in the Supplementary Data sheets Outcome_Mapping, Source_Numeric_Cells and Scope_Dispositions, rather than represented as completed duplicate verification.

Forty reports excluded at full text in an earlier phase were reassessed under the final construct definition, and four were subsequently included. The PubMed update identified 226 records absent from the historical export. After removal of four cross-database duplicates and one erratum or related record, 189 were excluded at title–abstract assessment and 32 reports were sought for full text. These 32 comprised 18 new inclusions, 10 exclusions, and four unretrieved reports. A separate identity-repair entry for an already included report did not belong to the 226-record incremental queue and was not counted as a thirty-third report in that pathway. Supplementary Table S3 and the screening ledger reconcile these stages.

The withdrawn Pinto-Escalona version was replaced by the formal version of record (Pinto-Escalona et al., 2024). Its four candidate effects remained outside quantitative pooling. Supplementary Table 4 in the source labels its means and standard deviations as adjusted, while raw means and standard deviations in Supplementary Table 5 cannot be linked to cell-specific sample sizes. Treating adjusted standard deviations as raw or inventing analyzed cell sizes would not provide a defensible variance. The withdrawn version remains only in the internal version history.

### 2.5 Data extraction, direction coding, and analytical classification

Data were organized at report, trial-family, and effect-estimate levels. Companion publications remained separate reports within the same family; four linked pairs account for the 74 reports and 70 families. For each candidate effect, extraction recorded the source location, instrument and psychological domain, informant, comparison and endpoint, scoring direction, sample-size basis, calculation method, cluster adjustment, and estimation assumptions.

Core outcomes represented direct social–emotional competencies; adjacent outcomes represented related psychological experiences or self-perceptions, as defined in the eligibility criteria. Classification followed instrument content rather than outcome labels alone. Positive effects indicated better functioning only where the measure had a defensible favorable direction; symptoms and difficulties were reversed accordingly. Outcomes without a uniformly favorable direction, contextual support measures, and mixed physical/psychological totals were kept separate from that pooled mean. Exclusion from this mean did not imply ineligibility for every construct-specific synthesis.

At the same endpoint and informant, an available total was selected over overlapping subscales; distinct instruments remained correlated outcomes, and combined-sex and sex-stratified estimates were not counted together. Outcomes were not selected by direction or statistical significance. These instrument-based refinements occurred during revision, not prospectively. Supplementary section S1 retains the study-specific decisions, and the Outcome_Mapping worksheet records instrument, informant, timing, scoring, classification, and unresolved source fields.

### 2.6 MMAT appraisal

All 74 reports were represented in the item-level Mixed Methods Appraisal Tool 2018 ledger (MMAT; Hong et al., 2018). Each report received two screening questions and five design-specific criteria; nine mixed-methods reports additionally received qualitative and integration criteria, yielding 608 item rows. Judgments were Yes, No, or Cannot tell, with source locations and reasons. No total score, percentage ranking, or quality threshold determined model eligibility. During revision, targeted source checks corrected ten item records, including a random-number cognitive task mistakenly cited as evidence of random allocation, an unrelated thesis chapter cited for recruitment, and teacher retention mistakenly used to support student outcome completeness. Remaining historical judgments and newly checked records are distinguished in the ledger; the targeted checks are not presented as a new independent appraisal of every item.

### 2.7 Effect-size calculation

Controlled effects were expressed as Hedges’ g with a small-sample correction, using standardization and sampling-variance calculations appropriate to the reported contrast (Hedges, 1981). Raw posttest means were standardized by the pooled within-group standard deviation; adjusted raw coefficients used an identified standardizer, and reported standardized coefficients retained their corresponding uncertainty. A raw posttest contrast is not a baseline-adjusted change effect merely because pretests were also collected (Morris, 2008). Some reconstructions depended on an inferred standardizer or conflicting source information, so even the source-informed set required conditional assumptions; declared sensitivities omitted the affected reports.

Uncontrolled within-group changes were estimated separately and do not measure intervention-versus-control effects (Morris and DeShon, 2002). Their variances depend on the pre–post correlation, distinct from the working correlation among effect estimates. Supplementary section S6 retains the formulas, standardizers, conditional Yan/Miller reconstructions, and Chan reporting discrepancy; Analysis_Routing records the source quantities, derivations, and assumptions. Source contradictions were retained as uncertainty rather than resolved by choosing a favorable value.

### 2.8 Trial-family dependence and multilevel models

Effects sharing participants, controls, arms, or endpoints were grouped by trial family. For distinct rows within a family, the working sampling covariance was rho multiplied by the square root of the two sampling variances; the diagonal contained the sampling variance itself. The primary working correlation was 0.60, with 0.30 and 0.80 sensitivities for core outcomes. This is a working approximation for correlated effect estimates, not a substitute for classroom or school clustering within a source study. Multilevel REML models included random intercepts at family and effect levels (Assink and Wibbelink, 2016), and family-clustered inference addressed residual dependence (Hedges et al., 2010). Neither procedure remedies treatment confounded with a single allocation cluster.

Models used rma.mv in metafor (Viechtbauer, 2010) and CR2 covariance estimation with Satterthwaite degrees of freedom (Tipton, 2015; Pustejovsky and Tipton, 2018). We report effect and family counts, estimates, CR2 standard errors, confidence intervals, degrees of freedom and *p* values, and both variance components. Approximate multilevel I^2^ divides total estimated heterogeneity by that heterogeneity plus the mean diagonal sampling variance; it is a descriptive, working-correlation-dependent summary, not an absolute effect-size range (Borenstein et al., 2017). Prediction intervals come from the fitted multilevel model using containment-based t inference in predict.rma.mv. They are not CR2 prediction intervals. They describe a model-implied distribution of effects in comparable contexts and are particularly unstable with three or five families (Higgins et al., 2009; IntHout et al., 2016).

The source-informed controlled set required a compatible contrast with reported allocation-aware uncertainty or a reconstructible correction using source-reported clustering information. The broader controlled set included this set plus effects requiring explicit external ICC or other variance assumptions. These are uncertainty-reconstruction categories, not rankings of overall methodological quality. Source-informed studies can retain substantial selection, measurement, or missing-data problems. Conversely, exclusion from that set can reflect incomplete reporting rather than poor conduct. The combined source-informed model and the broader model are nested; their *p* values were not compared as if they came from independent datasets.

### 2.9 Sensitivity, moderators, and reporting-bias analyses

Sensitivity analyses varied external ICCs over 0.01, 0.05, and 0.10; the working effect correlation of 0.60 was varied to 0.30 and 0.80 for core outcomes. Uncontrolled pre–post correlations were varied over 0.20, 0.50, and 0.80, retaining the available reported correlation. Allocation-aware eligibility and design-effect assumptions were applied consistently rather than selected by results. Revision analyses also examined broader outcome layers, alternative construct classification, total-versus-component representation, and source ambiguities. These were exploratory, source- or theory-based checks, not choices made by significance. Leave-one-family-out analyses described influence without automatically deleting families (Viechtbauer and Cheung, 2010).

Holm adjustment covered the core mean, adjacent mean, and adjacent-minus-core contrast; this testing family did not imply prospective confirmation (Holm, 1979). Domain coefficients and the two continuous moderators formed separate exploratory BH-FDR families (Benjamini and Hochberg, 1995). Tests were two-sided with 95% intervals and Holm-adjusted *p* < 0.05 for the three-test family. No common minimally important difference was established across the scales, and generic standardized-effect cutoffs were not used to infer practical benefit.

Routine funnel-asymmetry tests were unsuitable for the three- and five-family primary sets. The broader exploratory diagnostic related family-level composites to analyzed sample size, rather than treating correlated effects as independent or regressing standardized mean differences on potentially coupled standard errors (Pustejovsky and Rodgers, 2019). A sensitivity used nonnegative equal-weight family averages. These diagnostics cannot establish or exclude publication bias. Supplementary sections S10, S11 retain the design-effect implementation, study-specific omissions, diagnostic eligibility rules, GLS weighting, calculations, and source limitations.

### 2.10 Software and reproducibility

All comparisons were fitted in a fresh R 4.6.1 session using metafor 5.0-1 and clubSandwich 0.7.0, with seed 20260919. The submitted 19 models were refitted as explicitly labeled comparators, followed by 26 revision models and the declared influence and small-study analyses. The data workbook contains active revised inputs and outputs, while old results are isolated in a historical comparison sheet. Executed scripts, session information, model specifications, source-linked amendments, and input hashes accompany the revision materials.

## 3 Results

### 3.1 Study selection

The four-database union contained 924 records. Eighty duplicates and one erratum or related version were removed before screening, leaving 843 records. Of these, 712 were excluded at title or abstract. Full text was sought for 131 reports; 12 were not retrieved, 119 were assessed, and 45 were excluded with one primary reason per report. Seventy-four reports representing 70 trial families were included in the systematic review (Figure 1). Characteristics of the included reports are presented in Supplementary Table S4, and the complete full-text exclusion ledger is provided in the Supplementary Data workbook.

**Figure 1:**
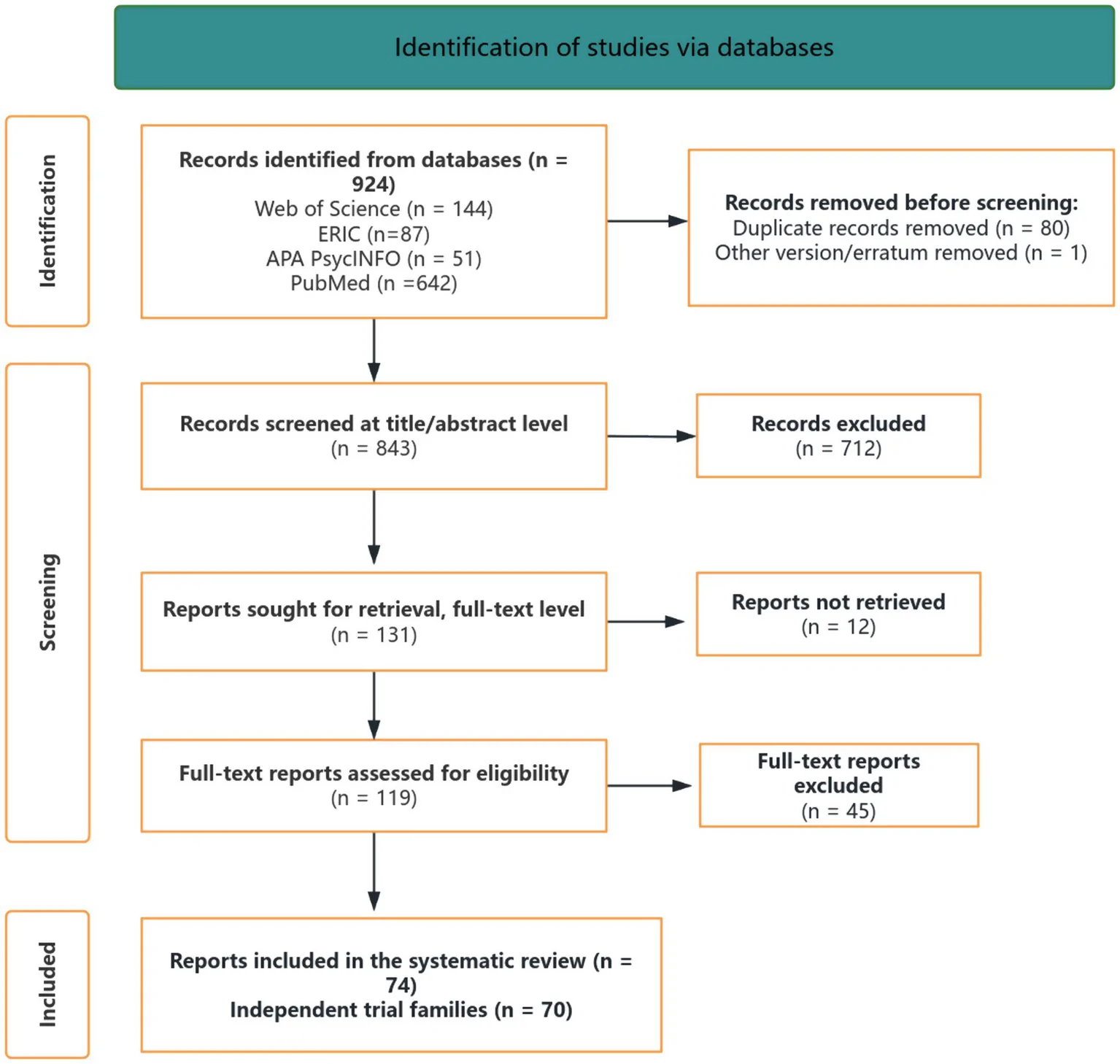
PRISMA 2020 flow diagram. Report retrieval, assessment, and inclusion are distinct stages; the separate PubMed identity-repair entry is not an additional report in the incremental retrieval pathway (Page et al., 2021).

### 3.2 Included reports, trial families, and analytical routes

The review retained 74 reports from 70 trial families. The integrated candidate register contained 316 records: 179 submitted rows, 34 added before the current source reconciliation, and 103 added subsequently. Candidate counts include descriptive and overlapping outcomes, not just effects eligible for pooling. The source-informed controlled set comprised 18 effects from eight families: five core effects from three families and 13 adjacent effects from five families. The nested broader set contained 126 effects from 28 families. Separately, 30 uncontrolled change effects from 6 families were analyzed. Twenty-two short-term between-group records from two families and two within-child contrasts from another family were described separately. The remaining routes comprised 89 numerically or design-ineligible records, 19 overlapping components, 7 physical-health records, 4 support-context records, and 17 other construct-specific descriptive records. Route family counts overlap and cannot be added to recover 70 (Table 1). Compared with the submitted analysis, two overlapping core components were removed; three adjacent records were moved to separate context or controlled-motivation categories and four source-supported adjacent outcomes were added. Thus, the submitted 7 core and 12 adjacent effects became 5 and 13, without changing the three and five contributing families (Supplementary section S12).

**Table 1:** Evidence sets and distinct counting units.

Evidence set	Effect records or reports	Trial families
Systematic review	74 reports	70
Candidate outcome register	316 records	See overlapping routes
Source-informed core	5	3
Source-informed adjacent	13	5
Combined source-informed	18	8
Broader controlled including primary	126	28
Uncontrolled pre–post	30	6
Short-term between-group records	22	2
Short-term within-child	2	1
Overlapping component records	19	6
Context perceptions separately described	4	2
Controlled regulation separately described	3	2
Not suitable for pooling	89	29
Other construct-specific descriptive records	14	7
Physical-health outcomes separately described	7	5

Family counts overlap across rows. Intervals refer to the stated model; individual effect counts do not measure independent information.

The primary core analysis drew on interventions assessing self-control, peer cooperation or empathy, and direct social–emotional competence (Condello et al., 2021; Pesce et al., 2021; Xu et al., 2025). A companion publication from the same cognitively engaging physical-activity trial provided additional report-level information but no additional primary effect (Xu et al., 2026). The primary adjacent analysis covered physical or game-related competence perceptions, motivation/confidence, enjoyment, and wellbeing across five independent families (Chan et al., 2019; Jimenez-Garcia et al., 2024; Miller et al., 2015; van Rossum et al., 2025; Yan et al., 2024). The recovered adjacent measures included autonomous motivation, movement-skill competence, and both reported game-perception factors; they were not relabeled as self-efficacy.

The broader controlled sensitivity analysis encompassed exergaming, mastery-climate motor development, dance, games-centred PE, enhanced PE, multisport programmes, yoga, cooperative or responsibility-oriented approaches, and gamification. Studies focused on self-esteem, perceived competence, motivation, social competence, and wellbeing (Berleze and Valentini, 2022; Borowiec et al., 2019; Cocca et al., 2020; Dallolio et al., 2016; Eckert et al., 2026; Fairclough et al., 2016). Additional programmes involved teacher- or class-level curricular changes, movement and dance instruction, and service-learning approaches (Fuller et al., 2017; Goñi and Zulaika, 2000; Invernizzi et al., 2022; Kuspratiwi et al., 2026; Likesas and Zachopoulou, 2006; Madsen et al., 2020; Mayorga-Vega et al., 2012). The remaining broader controlled reports extended this evidence to need-supportive, sociality-oriented, and motivational interventions (Park et al., 2017; Pozo Rosado et al., 2022; Sotos-Martínez et al., 2023). Recoverable need-supportive, motivational, dance, and gamified-resistance contrasts were additionally admitted under the same external-ICC rule (Abdulla et al., 2022; Chang et al., 2016; Vanaclocha-Amat et al., 2026; Vasilopoulos and Dumontheil, 2026). Yoga and dance contrasts shared the same usual-PE control and trial-family identifier (Vasilopoulos and Dumontheil, 2026). Their inclusion expanded coverage but retained explicit assumptions about allocation-aware variances, sparse clusters, and baseline comparability.

Uncontrolled pre-post reports evaluated running, tactical games, motor-competence and mental-health feasibility programmes, physical-activity cards, modified invasion games, healthy-lifestyle provision, and exergaming (Cakir et al., 2025; Fairclough et al., 2024; Harvey et al., 2017; Neira-Navarrete et al., 2024; Pippi et al., 2020; Pope et al., 2015; Xiang et al., 2006). Short-term designs were distinct: Burford et al. (2022) compared HIIT conditions within children, whereas Kolovelonis et al. (2013) randomized pupils between practice groups and reported posttest enjoyment and satisfaction. These sparse and design-specific records do not support a stable common short-term population estimate with family-level CR2 inference. Andrade et al. (2020) contributed immediate self-esteem and six mood-state domains, each reported separately by sex, after three lessons. These measures were described separately from the sustained controlled evidence; mood-state counts in the girls’ analysis did not reconcile with the table headers. The additional source inventory included intrinsic and identified motivation, amotivation, school and family wellbeing, and task values (Harvey et al., 2017; Pippi et al., 2020; Xiang et al., 2006). The source-reported total sample was retained without duplicating sex strata.

Report-level and structured qualitative evidence addressed confidence and resilience in Sport Education, self-regulation through martial arts, competition-related competence, emotional intelligence, cooperation, respect, social responsibility, and teacher- or team-teaching practices (Ang and Penney, 2013; Çalık et al., 2018; Castle and Howells, 2024; Lakes and Hoyt, 2004; Rice et al., 2025; Rivera-Pérez et al., 2021). Further qualitative or feasibility evidence covered cooperative learning, gamified PE, social–emotional learning, and prosocial development (Russo et al., 2025; Ryom et al., 2022; Sindiani et al., 2025; Xie et al., 2025).

Eligible psychological results were not pooled when the allocation or analysis unit could not be aligned, treatment was confounded with cluster, sampling variance was unavailable, the estimand was incompatible, reporting was internally inconsistent, nonparametric statistics were insufficient, or covariance from shared controls could not be resolved. Remaining limitations affected reports on short TGfU exposure, active gamification, dramatization, autonomy support, and fundamental motor skills (Arias-Estero et al., 2020; Carcelén-Fraile, 2025a; Carcelén-Fraile et al., 2025b; Castillo-Viera et al., 2021; Chang et al., 2016; Chen et al., 2026). Similar limitations affected neuromuscular training, yoga, questioning in TGfU, enriched sport programmes, appropriate-behavior PE, autonomy-supportive teaching, and emotional wellbeing (Duncan et al., 2018; Folleto et al., 2016; Gaspar et al., 2021; Gentile et al., 2020; Gil-Madrona et al., 2019; Huéscar Hernández et al., 2020; Kliziene et al., 2021). Other affected reports involved team-building, Sport Education, team teaching, exergame framing, emotional-growth programmes, karate, rhythmic teaching, and impulse control (Liu et al., 2025; Luna et al., 2020; Luna et al., 2021; Luptáková et al., 2025; Lwin and Malik, 2014; Mira et al., 2026; Pinto-Escalona et al., 2024). The remaining non-pooled reports addressed developmental coordination, creative movement, creative dance, and the companion mental-health report from the cognitively engaging activity trial (Rigon et al., 2024; Rodríguez-Negro and Yanci, 2022; Sit et al., 2019; Theodorakou and Zervas, 2003; Vasilopoulos and Dumontheil, 2026; Xu et al., 2026). Some reports contributed recoverable effects while other measures remained unpooled. Supplementary Table S8 summarizes the affected reports, Supplementary Table S9 groups the reasons for non-pooling, and the Supplementary Data workbook provides the complete effect-level record.

Mira’s two historical effect identifiers were retained as aliases of the same report, not new reports. Its 41 enrolled versus 38 paired intervention observations could not be assigned unambiguously to every descriptive cell. Treatment was confounded with grade (Ruiz-Montero et al., 2025), and Nigg (2024) did not report outcome-specific analyzed arm sizes; those contrasts were withheld rather than repaired by assumed clustering or inferred sample sizes. Pope’s psychological endpoint denominator was likewise insufficiently identified for the revised uncontrolled analysis.

Additional descriptive mapping distinguished the three TGfU domains of enjoyment, perceived competence and intention from a single psychosocial score (Arias-Estero et al., 2020). In the uncontrolled yoga report, questionnaire-based cognitive/motor competence and social/maternal acceptance did not show clear changes, whereas interviews described calmness and enjoyment (Folleto et al., 2016). These were different measures, not interchangeable estimates. The five teaching configurations in Luptáková et al. (2025) had no common untreated comparator. Lwin and Malik (2014) crossed PE format with message framing; both framing strata and all six beliefs were retained in the descriptive inventory. The Carcelén-Fraile (2025a) table included decreases in appropriate social skills and increases in impulsiveness, alongside source-scoring inconsistencies; its findings were not uniformly coded as beneficial. Supplementary Data provides the domain- and configuration-specific dispositions.

### 3.3 Methodological appraisal

The ledger comprised 148 screening questions and 460 design-specific items. After the targeted source corrections, randomized-trial items were 35 Yes, two No, and 138 Cannot tell; the other category counts are given in Supplementary Table S5. Counts were not converted into a quality score. The primary evidence retained important limitations: Miller reported baseline imbalances; Pesce excluded incomplete cases; Yan’s valid questionnaire denominator differed from its table headings; and Jimenez-Garcia’s convenience recruitment restricted representativeness. Source-informed uncertainty therefore did not imply low risk of bias. Cannot tell indicates insufficient or conflicting reporting, not a demonstrated high- or low-risk condition. No GRADE certainty assessment was performed.

### 3.4 Core social–emotional outcome model

The revised core model contained five effects from three families (Figure 2): g = 0.947, 95% CI [−0.774, 2.667], CR2 SE = 0.399, Satterthwaite df = 2.00, *p* = 0.141, Holm-adjusted *p* = 0.424. Its model-based prediction interval was [−2.387, 4.280] (Table 2). Total tau^2^ was 0.436 and approximate I^2^ was 87.42%; variance components and heterogeneity summaries are provided in the Model_Results_R1 worksheet of the Supplementary Data workbook. Selecting Xu’s total instead of total-plus-components reduced the effect-row count without increasing the independent information. The estimate was imprecise and both intervals included unfavorable and favorable values.

**Figure 2:**
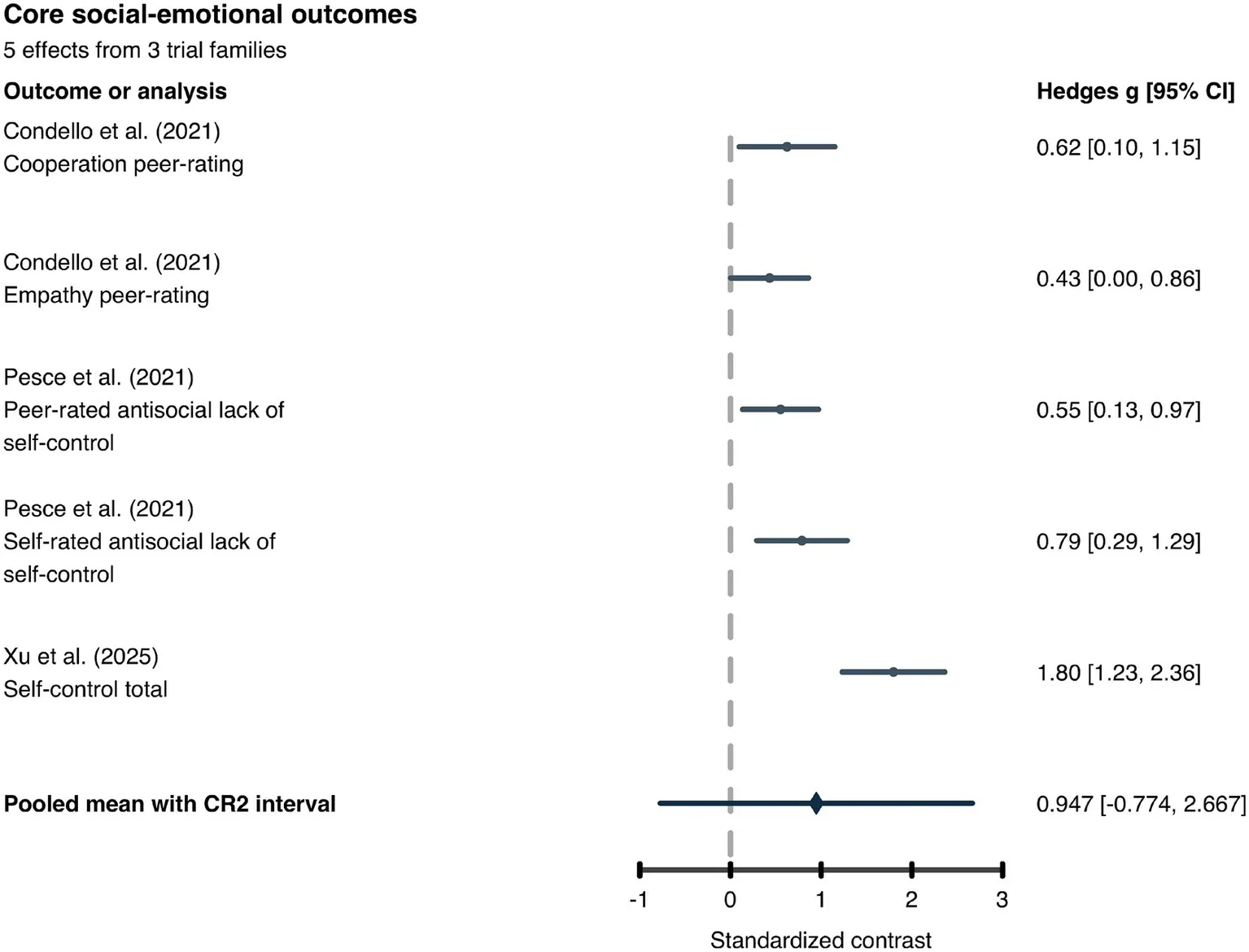
Source-informed core effects. Points show Hedges’ g and normal-approximation 95% intervals from the extracted sampling variances; correlated rows are not independent trials. The diamond marks the pooled mean with a CR2/Satterthwaite 95% confidence interval. Higher values favor psychological functioning. The model-based prediction interval is reported in Table 2, not represented by the diamond.

**Table 2:** Revised model estimates and uncertainty.

Analysis	Effects/families	g [95% CR2 CI]	SE / df	Raw p	Holm p	Model PI
Core source-informed	5/3	0.947 [−0.774, 2.667]	0.399/2.00	= 0.141	= 0.424	[−2.387, 4.280]
Adjacent source-informed	13/5	0.215 [−0.386, 0.817]	0.205/3.51	= 0.360	= 0.424	[−1.368, 1.799]
Combined source-informed	18/8	0.421 [−0.001, 0.843]	0.175/6.35	= 0.0504	Not applied	[−0.984, 1.826]
Broader controlled	126/28	0.381 [0.135, 0.627]	0.120/26.73	= 0.004	Not applied	[−0.913, 1.676]
Broader core	15/7	1.024 [−0.074, 2.122]	0.447/5.93	= 0.063	Not applied	[−2.015, 4.062]
Broader adjacent	111/24	0.359 [0.102, 0.615]	0.124/22.69	= 0.008	Not applied	[−0.886, 1.603]
Uncontrolled change	30/6	0.128 [−0.377, 0.632]	0.196/4.99	= 0.544	Not applied	[−1.220, 1.475]

CI, confidence interval; PI, model-based prediction interval. Sensitivity analyses are exploratory. No overall MMAT quality score determined these sets.

### 3.5 Adjacent psychological outcome model

The adjacent model included 13 effects from five families (Figure 3): g = 0.215, 95% CI [−0.386, 0.817], CR2 SE = 0.205, Satterthwaite df = 3.51, *p* = 0.360, Holm-adjusted *p* = 0.424. Its model-based prediction interval was [−1.368, 1.799], total tau^2^ = 0.286, and approximate I^2^ = 88.97%. Physical self-perceptions, motivation/confidence, autonomous and intrinsic motivation, enjoyment, and wellbeing contributed to this mean. Teacher support and controlled regulation did not enter this pooled favorable-direction estimate.

**Figure 3:**
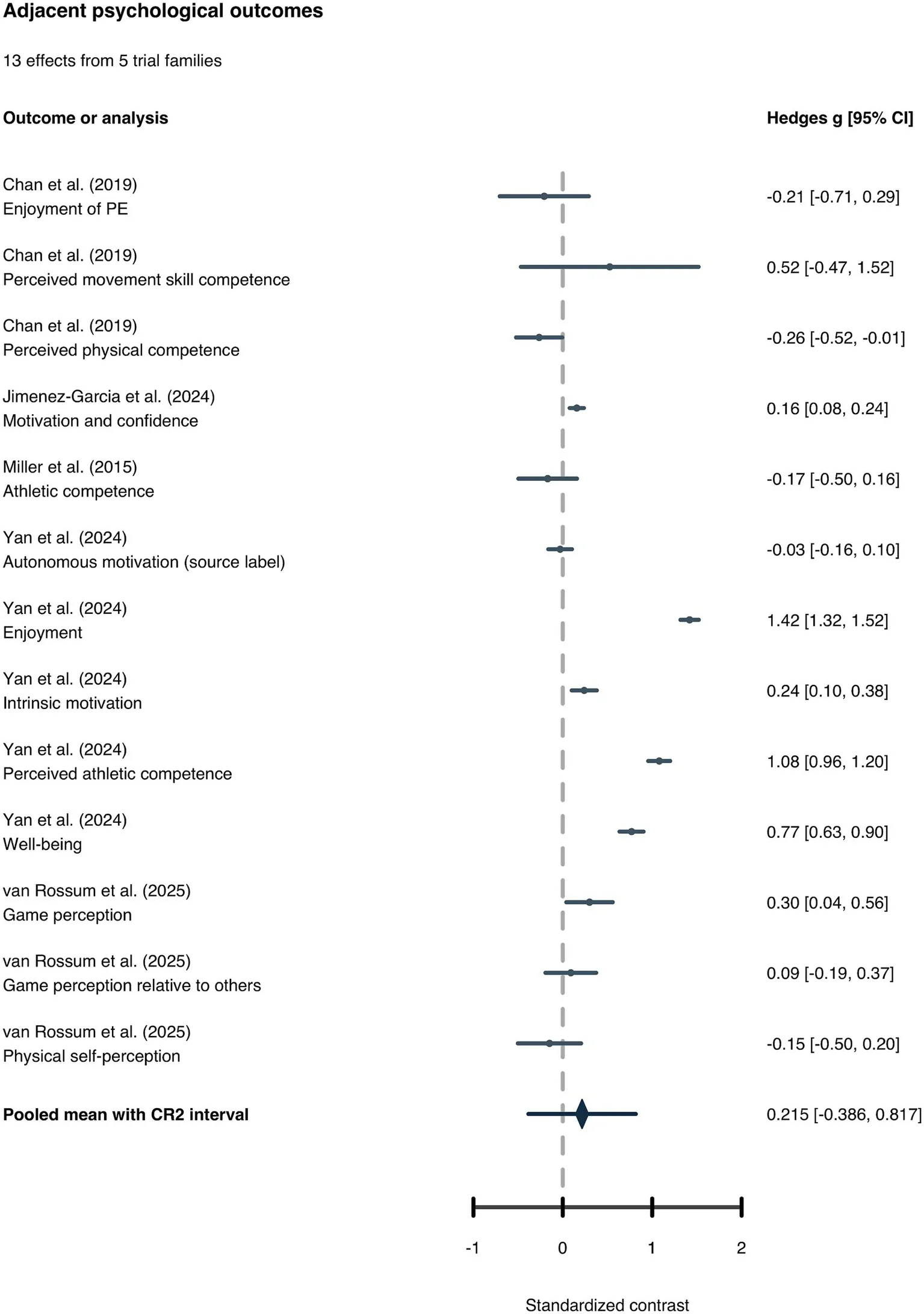
Source-informed adjacent effects. Points and lines are individual Hedges’ g estimates and normal-approximation 95% intervals. The pooled interval is CR2/Satterthwaite. Perceived competence, self-perception, and confidence retain their source-specific meaning and are not relabeled self-efficacy. Teacher support and controlled regulation are described separately. Source-label and coefficient discrepancies are documented in the data workbook.

### 3.6 Direct layer comparison and construct-level analyses

The combined source-informed set yielded g = 0.421, 95% CI [−0.001, 0.843], CR2 SE = 0.175, Satterthwaite df = 6.35, *p* = 0.0504. The adjacent-minus-core coefficient was b = −0.688, CR2 SE = 0.383, df = 3.67, 95% CI [−1.789, 0.413], raw *p* = 0.153, and Holm-adjusted *p* = 0.424. The layer coefficient is a between-family observational comparison, not a randomized test that one psychological construct responds more strongly than another.

Exploratory domain contrasts were imprecise, with very low denominator degrees of freedom. None was supported after BH-FDR adjustment; this did not establish equivalence of domains. Publication-year and log-sample-size coefficients likewise remained uncertain. Full coefficients, raw and adjusted *p* values, and degrees of freedom are provided in the Supplementary Data workbook.

Table 2 reports primary and broader model estimates, including their different inferential assumptions.

### 3.7 Heterogeneity and robustness analyses

The broader controlled set yielded g = 0.381, 95% CI [0.135, 0.627], CR2 SE = 0.120, Satterthwaite df = 26.73, *p* = 0.004, with prediction interval [−0.913, 1.676] (Figure 4). The added-assumption subset comprised 108 effects from 21 families; family membership partly overlapped the source-informed set. Under external ICCs of 0.01, 0.05, and 0.10, pooled means were 0.384, 0.381, and 0.379. Broader core and adjacent means were g = 1.024, 95% CI [−0.074, 2.122] and g = 0.359, 95% CI [0.102, 0.615], respectively. These layers shared some families. The broader mean and combined source-informed mean differed little numerically, but the broader interval was narrower; their different *p* values do not establish contradictory findings or equivalence. Omitting the three reports with the declared source ambiguities left 95 effects from 25 families and yielded g = 0.338, 95% CI [0.142, 0.534]. This checks sensitivity to those reports; it does not establish that the remaining reports are free of bias.

**Figure 4:**
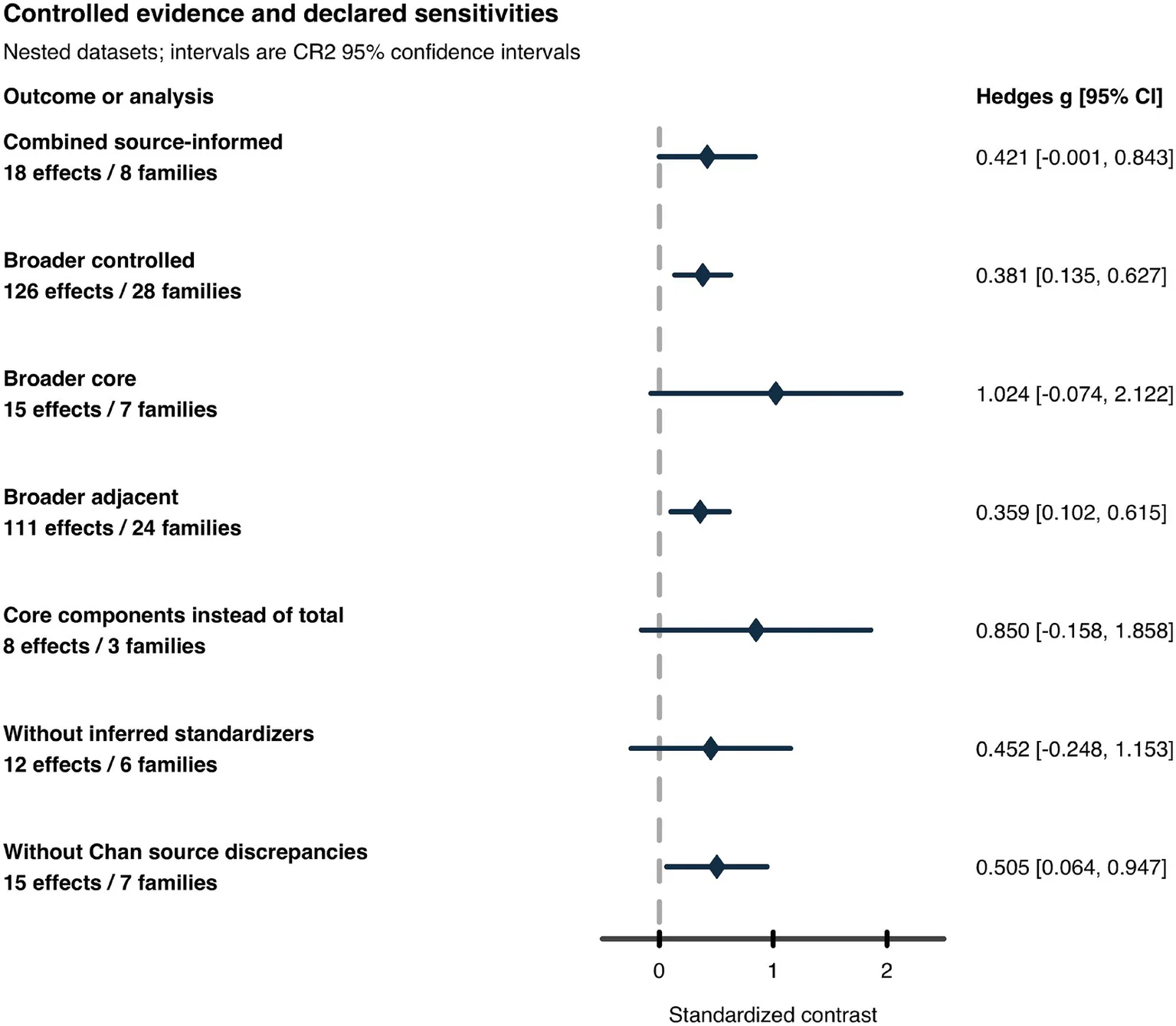
Controlled evidence sets and declared sensitivities. All displayed intervals use CR2/Satterthwaite inference. Sets overlap and are not statistically independent. Broader models add explicit variance and clustering assumptions, not a uniform low-quality category. No sensitivity was selected as the preferred result based on significance.

The separate uncontrolled analysis yielded g = 0.128, 95% CI [−0.377, 0.632], CR2 SE = 0.196, Satterthwaite df = 4.99, *p* = 0.544 across 30 effects and 6 families. Its estimates under pre–post correlations 0.20 and 0.80 were 0.133 and 0.122. These are changes without a concurrent comparator. Omitting Neira-Navarrete yielded g = 0.138, 95% CI [−0.532, 0.807]. The short-term evidence was described without a combined pooled effect. Component-replacement, alternative-taxonomy and source-ambiguity estimates appear in Table 3 and Supplementary Table S7; none replaced the main analysis according to statistical significance.

**Table 3:** Declared exploratory sensitivity analyses.

Exploratory analysis	Effects/families	Estimate [95% CR2 CI]	p
Core, working correlation 0.30	5/3	0.953 [−0.738, 2.645]	= 0.136
Core, working correlation 0.80	5/3	0.947 [−0.775, 2.668]	= 0.142
Broader, external ICC 0.01	126/28	0.384 [0.138, 0.630]	= 0.004
Broader, external ICC 0.10	126/28	0.379 [0.134, 0.625]	= 0.004
Core, all components instead of total	8/3	0.850 [−0.158, 1.858]	= 0.067
Alternative taxonomy, agency/core	11/8	0.437 [−0.129, 1.002]	= 0.111
Alternative taxonomy, adjacent	7/3	0.385 [−0.983, 1.753]	= 0.270
Combined, without inferred standardizers	12/6	0.452 [−0.248, 1.153]	= 0.158
Combined, without Chan report	15/7	0.505 [0.064, 0.947]	= 0.032
Broader, source-ambiguity omission	95/25	0.338 [0.142, 0.534]	= 0.002
Uncontrolled, without Neira report	24/5	0.138 [−0.532, 0.807]	= 0.599

CI, confidence interval; PI, model-based prediction interval. Sensitivity analyses are exploratory. No overall MMAT quality score determined these sets.

Holm adjustment covers the three main tests. BH-FDR applies only to the stated exploratory moderator families. Other sensitivity estimates are descriptive and exploratory.

Core leave-one-family-out means ranged from 0.553 to 1.196, with only two families remaining in each model. These describe influence, not established robustness. In the broader exploratory sample-size analysis, 22 of 28 families had one consistent pair of analyzed arm sizes. The sample-size predictor coefficient was 3.085 (SE = 2.141; 95% CI [−1.382, 7.552]; *p* = 0.165). The interval included zero, so this diagnostic did not establish a size association. GLS assigned negative weights to 2 outcomes in 2 families. The nonnegative equal-weight sensitivity gave 2.937, 95% CI [−1.461, 7.334], *p* = 0.179. These results cannot exclude publication bias or distinguish selective reporting from differences in design, constructs and context.

Table 3 summarizes the declared sensitivity analyses; full moderator results are supplied in the Supplementary Data workbook.

## 4 Discussion

### 4.1 Principal findings

Only three and five independent families informed the core and adjacent source-informed estimates, despite the wider report-level evidence base. Their wide confidence and prediction intervals leave both average effects and their distribution uncertain. The combined source-informed estimate (g = 0.421) and broader controlled estimate (g = 0.381) both point toward a possible average benefit, with only the latter estimated precisely enough for its confidence interval to exclude zero. This agreement is encouraging, but the analyses share evidence and the broader estimate relies on additional assumptions; substantial heterogeneity also limits predictions for individual schools and outcomes.

This conclusion is compatible with, but more circumscribed than, broader syntheses. Universal school-based social–emotional programs can improve developmental outcomes when instruction is explicit, sequenced, active, and focused (Durlak et al., 2011; Taylor et al., 2017). Reviews of physical-activity and combined physical-activity–social–emotional programs also identify promising findings, alongside marked variation in setting, program content, age, measurement, and study quality (Jackson et al., 2024; Moon et al., 2024). By isolating curricular PE, distinguishing direct social–emotional competencies from adjacent psychological outcomes, and retaining dependence among estimates, this review defines a narrower inferential target. Its cautious conclusion concerns the limits of the available evidence rather than evidence that psychological benefits cannot occur.

The DE-PASS findings reinforce a separate boundary: a change in a psychological determinant need not be accompanied by measurable behavior change. Khudair et al. (2025) found generally limited effects across device-measured activity or sedentary outcomes and their determinants, varying by setting. Ling et al. (2024) identified instances of determinant change without corresponding behavioral improvement and highlighted the limited mediation evidence. These reviews do not show that PE changes, or fails to change, core social–emotional competence. They instead caution against assuming that a theoretical target, intervention exposure, psychological measure, and distal behavior necessarily form a demonstrated causal chain.

### 4.2 Interpretation across psychological outcome domains

Emotion regulation and self-control are plausible targets in PE because lessons can evoke frustration, excitement, competition, social evaluation, and rapid decisions. These experiences may provide occasions to recognize emotion, pause, select a response, or recover from error, but exposure is not equivalent to learning. Emotion-regulation processes are distinguishable, and self-control can involve proactive management of situations as well as effortful inhibition (Gross, 2015; Duckworth and Steinberg, 2015; Duckworth et al., 2016). An intervention must therefore specify the intended process, provide guided practice and feedback, and assess an outcome that corresponds to that process. The imprecise core point estimate cannot be interpreted as proof of a substantial transferable benefit, especially when only three families informed the model and operationalizations differed across studies.

Empathy, prosocial competence, and relationship skills likewise require more than physical co-presence. Cooperative and responsibility-oriented tasks can create shared goals, interdependence, perspective taking, and opportunities to manage conflict when roles, accountability, facilitation, and reflection are intentionally designed. Existing reviews nevertheless describe uneven affective evidence, incomplete follow-up, and uncertainty about transfer and active ingredients (Casey and Goodyear, 2015; Pozo et al., 2018; Dyson et al., 2021). The exploratory domain analysis did not identify reliable differences after false-discovery-rate correction, but sparse family cells and low CR2 degrees of freedom preclude interpreting this as evidence of equivalence or absence of moderation (Hedges and Pigott, 2004).

Adjacent outcomes also require measure-specific interpretation. The primary studies chiefly assessed athletic or physical self-perceptions, pictorial competence, game-related perceptions, motivation/confidence, enjoyment, motivational regulation, and wellbeing. They did not all measure task-specific self-efficacy. This matters because a child may become more accurately calibrated about skill while reporting lower perceived competence, or may enjoy a lesson without gaining broader self-regulation. Need-supportive environments offer a plausible motivational account, but these pooled outcomes do not test that pathway (Bandura, 1977; Ryan and Deci, 2020; Vasconcellos et al., 2020). The alternative self-awareness/agency classification redistributed evidence across layers, illustrating the dependence of category summaries on the operational framework rather than identifying a uniquely correct boundary.

### 4.3 Pedagogical and implementation implications

The evidence does not justify ranking named pedagogical approaches. It does support more specific design questions. In the primary sources, enriched PE explicitly targeted self-control and peer behavior, while teacher-development approaches targeted motivational climate and game learning (Pesce et al., 2021; Condello et al., 2021; Yan et al., 2024). These targets should not be interpreted as interchangeable mechanisms. In Pozo Rosado et al. (2022), using the empathy total instead of a selected favorable component also showed why recommendations should not be built around whichever subscale was statistically supported. Cooperation, responsibility, and reflection therefore remain priorities for theory-driven intervention research (Casey and Goodyear, 2015; Pozo et al., 2018; Dyson et al., 2021).

Teacher support should be treated as an intervention process to be measured alongside, rather than substituted for, a student outcome. Chan et al. (2019) reported more perceived teacher support alongside an unfavorable adjusted physical-competence contrast; other competence and enjoyment estimates were uncertain. In van Rossum et al. (2025), game-related perception and physical self-perception also differed, while observations documented limited individual movement feedback. Burford et al. (2022) reported greater enjoyment and perceived competence under more autonomous HIIT conditions alongside lower vigorous activity. These patterns argue for evaluating intended benefits and trade-offs using construct-matched measures, rather than assuming every desirable feature improves every outcome.

Implementation evidence is necessary to determine whether an unsuccessful result reflects the program theory, its delivery, or both. Fidelity assessment should document exposure, adherence, quality of delivery, participant responsiveness, and relevant adaptations rather than relying on the intervention label alone (Carroll et al., 2007; Durlak and DuPre, 2008). The present data did not provide complete, verified moderator information for cooperation, responsibility, reflection, teacher behavior, dosage, or fidelity. These features should therefore be viewed as testable design propositions, not as mediators established by this meta-analysis.

### 4.4 Methodological implications

The review illustrates why report, trial-family, and effect levels must remain distinct. Multiple publications can arise from the same intervention, and a single report can contribute several outcomes, arms, or endpoints. Treating these rows as independent studies would overstate the information available for inference. The multilevel models and trial-family cluster-robust standard errors addressed dependence, but robust estimation cannot create independent information when only three or five families inform a pooled estimate. Family counts and small-sample degrees of freedom are therefore as important as the number of effect estimates.

Approximate I^2^ remained high (87.42% core; 88.97% adjacent), but a pooled random-effects mean is not premised on one identical intervention effect. It estimates an average over the represented distribution of programs, outcomes, and settings. That average has limited substantive meaning when the mix is poorly characterized and is not a prediction for a particular school. The wide model-based prediction intervals underscore this problem, while their own instability with few families prevents treating their endpoints as firmly established bounds (Borenstein et al., 2017; IntHout et al., 2016). Informant, measurement, baseline differences, comparator content, duration, and implementation may contribute to variation. Sparse moderator cells and low degrees of freedom cannot establish that these factors are unimportant.

The revised broader analysis increased coverage through explicit, consistently applied assumptions, including recoverable contrasts with two allocation clusters per arm. Its positive result should not be discounted as evidence from universally low-quality studies. Nevertheless, an assumed ICC does not repair baseline confounding, uncertain reporting, or implementation differences. Likewise, the source-informed set is not automatically trustworthy on every bias domain: some standardizers were inferred and some sources contained contradictions. The revised exploratory sample-size diagnostic was inconclusive; its null-compatible interval does not rule out missing evidence or selective reporting. The appropriate synthesis therefore presents both sets, their common signal and unequal precision, and the assumptions each requires.

### 4.5 Strengths and limitations

Strengths include separating reports, trial families, and effect estimates; preserving source-linked amendments; avoiding total–component duplication; and distinguishing short-term designs. The reconciled PubMed ledger and comparison with submitted analyses make the evidence pathway and revision decisions traceable.

Review-process limitations remain. The review was not prospectively registered. The authors recall planning the review, but contemporaneous documents cannot be recovered, so their original content and timing relative to searching, screening, and extraction cannot be verified. Later decisions are therefore not pre-data confirmation. English-only eligibility and unrecovered historical search details may limit coverage and reproducibility; the documented PubMed rerun cannot recreate undocumented historical behavior. One author performed the initial screening, extraction, classification, and appraisal. Subsequent author verification was not independent duplicate review and cannot fully remove correlated errors or confirmation bias. Supplementary sections S2, S10 document the search and revision-checking procedures.

The evidence itself limits inference. Only three and five independent families supported the core and adjacent analyses, respectively, and heterogeneous constructs and contexts restrict the meaning of an average effect. Source inconsistencies and conditional reconstructions add uncertainty that sensitivity analyses can address only partly. Supplementary sections S8–S11 and the Source_Numeric_Cells and Scope_Dispositions worksheets retain specific discrepancies, non-pooling reasons, and verification gaps. Unpooled outcomes are not zero effects. Instrument-level and participant-overlap checks were limited in scope; completed checks and unresolved issues are documented in Supplementary section S11.

Primary-study biases also affect confidence in both controlled sets. Uncertain allocation and baseline imbalance permit confounding; incomplete outcomes permit selective retention; and self-report or unblinded assessment permits measurement bias. An MMAT “Cannot tell” judgment means insufficient reporting, not that a criterion was met or that bias definitely occurred. Source-informed uncertainty is not equivalent to low risk of bias, and a positive broader average is not a high-certainty estimate. No GRADE certainty assessment was performed. Item-level MMAT judgments remain available without an overall quality score (Supplementary section S5 and MMAT_Item_Level).

### 4.6 Future research

Future work should first increase the number of independently allocated schools or classes with adequate cluster-aware planning. Next, trials should prespecify constructs and primary measures, provide analyzed arm and cluster counts, report adjusted effects with matching uncertainty and standardizers, and document total–subscale relationships. Outcome-level missingness, shared controls, and participant overlap across companion reports should be explicit. These steps would address the particular sources of imprecision and avoidable exclusions encountered here before adding more exploratory moderator analyses.

Intervention development should connect each proposed mechanism to observable pedagogy and temporally ordered measurement. A study testing autonomy support, cooperation, responsibility, reflection, or feedback should document how that feature was delivered, assess participant exposure and responsiveness, measure the hypothesized mediator, and use a construct-matched outcome. Longer follow-up and evidence from students, teachers, peers, or observers would help determine whether lesson-specific motivation or perceived competence transfers to durable emotion regulation, self-control, prosocial competence, or wellbeing. Adequately powered replications across schools and contexts are needed before particular pedagogical features can be identified as reliable moderators.

## 5 Conclusion

Primary-school PE interventions may support psychological functioning, and the broader controlled evidence showed a positive average. However, only three and five independent families informed the source-informed core and adjacent estimates, which remained imprecise. The broader and combined primary point estimates were similar; different confidence intervals or *p* values did not establish a substantive disagreement. Heterogeneous outcomes, additional variance assumptions, source inconsistencies, and limited sensitivity of small-study diagnostics limit generalization. The evidence does not demonstrate absence of benefit, identify universally effective pedagogy, or establish psychological mechanisms. More independent trials with aligned targets, implementation measures, and defensible uncertainty estimates are needed.

## Data Availability

The data extracted from published studies and the analysis code are provided in the Supplementary material. Further inquires can be directed to the corresponding author.

## Supplementary material

SUPPLEMENTARY Table 1 Sections S1-S12, including methodological details and supplementary tables.

SUPPLEMENTARY FILE 1 Extracted study data, study characteristics, outcome classifications, methodological appraisal, analysis routing, model results, and revision crosswalks.

SUPPLEMENTARY FILE 2 R analysis scripts, input datasets, model specifications, saved results, session information, and records documenting the input-file versions.
