# Supplementary material for: Psychological outcomes of primary-school physical education interventions: a systematic review and multilevel meta-analysis
Source: Front Psychol. 2026 Oct 7;17:1962805. doi: 10.3389/fpsyg.2026.1962805 (PMC13683171; doi:10.3389/fpsyg.2026.1962805)
Supplement: Supplementary file 1 [file Table_1.DOCX]

Supplementary Material

This file provides the search methods, study characteristics, methodological appraisal, and statistical details that support interpretation and reproducibility of the review.

# S1. Outcome taxonomy and analysis sets

For outcomes with a defensible favorable direction, positive effects indicate better functioning in the intervention condition; symptoms, difficulties, and dysregulation were reversed where the scale meaning supported this. Introjected and external regulation were retained in their original direction and described separately: neither an increase nor a decrease was automatically treated as a universal psychological benefit. Instrument content distinguished students’ subjective experience from exposure to support. School-, peer- and family-related wellbeing were adjacent psychological outcomes when items assessed experience, satisfaction, belonging or worry; teacher encouragement or instructional support was described separately. Physical-health HRQoL and mixed physical/psychological totals were outside the cross-construct psychological mean. The KINDL school subscale was consequently retained, rather than excluded because its name referred to school (Pippi et al., 2020). SPPC behavioral conduct was treated as behavioral self-perception, not demonstrated skill (Theodorakou and Zervas, 2003).

At a common endpoint and informant, an available total score was selected over overlapping components. Thus, Xu’s self-control total was not pooled together with its social-interaction and emotional-experience components; a sensitivity model replaced it with all four recoverable components. The same rule was applied to Pozo Rosado’s total/component measures and Fairclough’s SDQ total difficulties. SDQ prosocial behavior was retained because it is not part of the difficulties total. Distinct instruments measuring related constructs remained correlated outcomes, not assumed duplicates. Newly located eligible outcomes were appended with new effect identifiers, and were not selected according to their direction or statistical significance. The Outcome_Mapping worksheet identifies instrument, informant, timing, scoring, classification, and unresolved source fields.

Pictorial social acceptance was classified as a self-perception rather than demonstrated social competence. Abdulla’s additional need-frustration, autonomous-motivation, amotivation and self-efficacy outcomes followed the same variance rules as its previously extracted outcomes. Composite means and variances were not invented where only subscales were reported. Its teacher-rated engagement was withheld because the reported total and arm statistics did not reconcile. Andrade’s immediate mood and self-esteem responses after three lessons were described as short-term outcomes, rather than sustained intervention effects; unavailable sex-specific clustering and inconsistent mood denominators were not reconstructed from nominal headers.

These refinements were made during revision after examining the instruments, not prespecified before the review. Intrinsic and identified regulation, mastery-oriented motivation and psychological task values were distinguished from controlled regulation, ego/work-avoidance orientation and future behavioral intention. The latter were retained in source direction for construct-specific description. Exclusion from a uniformly favorable cross-construct mean does not imply that no construct-specific quantitative synthesis could ever be appropriate. No extra model was imposed where independent comparable evidence was too sparse. Separately measured global self-worth was not mistaken for the sum of domain subscales; combined-sex and sex-stratified estimates were not counted together. The taxonomy is operational and the instrument-level mapping identifies remaining verification gaps explicitly.

Supplementary Table S1. Evidence sets and units.

| Evidence set | Effect records or reports | Trial families |
| --- | --- | --- |
| Systematic review | 74 reports | 70 |
| Candidate outcome register | 316 records | See overlapping routes |
| Source-informed core | 5 | 3 |
| Source-informed adjacent | 13 | 5 |
| Combined source-informed | 18 | 8 |
| Broader controlled including primary | 126 | 28 |
| Uncontrolled pre–post | 30 | 6 |
| Short-term between-group records | 22 | 2 |
| Short-term within-child | 2 | 1 |
| Overlapping component records | 19 | 6 |
| Context perceptions separately described | 4 | 2 |
| Controlled regulation separately described | 3 | 2 |
| Not suitable for pooling | 89 | 29 |
| Other construct-specific descriptive records | 14 | 7 |
| Physical-health outcomes separately described | 7 | 5 |

# S2. Search strategies and PubMed reproducibility rerun

The exact historical PubMed query and run date were not recovered. The selected high-sensitivity replacement query was executed on 30 July 2026 with the publication-date cutoff fixed at 31 May 2026; it returned 513 records and retrieved all 11 prespecified sentinel reports. The rerun shared 287 PMIDs with the historical 416-PMID export. The 226 rerun-only records were assessed using the same eligibility criteria. The incremental queue comprised 32 reports sought: 18 included, 10 excluded, and four unretrieved. One separate already-included identity repair is outside that queue.

Supplementary Table S2. Database coverage and search verification.

| Database | Date | Coverage | Limits | Verification |
| --- | --- | --- | --- | --- |
| Web of Science Core Collection | 2026-05-28 | January 2000–May 2026 | English; peer-reviewed journal articles | Exact string recovered from archived supplementary material |
| ERIC | Exact run date not recovered; archived range ends 2026-05 | January 2000–May 2026 | English; peer-reviewed journal articles | Exact string recovered from archived supplementary material |
| APA PsycINFO via ProQuest | 2026-05-29 | January 2000–May 2026 | English; peer-reviewed journal articles | Exact string recovered from archived supplementary material |
| PubMed | 2026-07-30 actual rerun | 2000-01-01 to 2026-05-31 | No language filter; query fields as displayed | Original historical query not recovered; reproducible selected rerun executed through NCBI E-utilities; 513 records; 11/11 sentinels. |

## S2.1 Web of Science Core Collection exact strategy

TS=("physical education" OR "PE class*" OR "sport education" OR "movement education" OR "physical activity") AND TS=("emotional intelligence" OR "emotional competence" OR "emotional awareness" OR "emotional regulation" OR empathy OR "self-esteem" OR "self-control" OR "social skill*" OR prosocial OR motivation) AND TS=(intervention OR program* OR trial OR experiment* OR "controlled") AND TS=("primary school" OR "elementary school" OR child* OR pupil*)

## S2.2 ERIC exact strategy

("physical education" OR "PE class" OR "sport education" OR "movement education" OR "physical activity") AND ("emotional intelligence" OR "emotional competence" OR "emotional regulation" OR empathy OR "self-esteem" OR "self-control" OR "social skills" OR prosocial OR motivation) AND (intervention OR program OR trial OR experiment) AND ("primary school" OR "elementary school" OR children OR pupils)

## S2.3 APA PsycINFO via ProQuest exact strategy

("physical education" OR "PE class" OR "sport education" OR "movement education" OR "physical activity") AND ("emotional intelligence" OR "emotional competence" OR "emotional awareness" OR "emotional regulation" OR empathy OR "self-esteem" OR "self-control" OR "social skills" OR prosocial OR motivation) AND (intervention OR program OR trial OR experiment) AND ("primary school" OR "elementary school" OR children OR pupils)

## S2.4 PubMed exact strategy

(
 "Physical Education and Training"[Mesh]
 OR "physical education"[Title/Abstract]
 OR "PE class"[Title/Abstract]
 OR "PE classes"[Title/Abstract]
 OR "sport education"[Title/Abstract]
 OR "movement education"[Title/Abstract]
 OR "physical education lesson"[Title/Abstract]
 OR "physical education lessons"[Title/Abstract]
)
AND
(
 "social emotional learning"[Title/Abstract]
 OR "social-emotional learning"[Title/Abstract]
 OR "social emotional competence"[Title/Abstract]
 OR "social-emotional competence"[Title/Abstract]
 OR "emotional intelligence"[Title/Abstract]
 OR "emotional competence"[Title/Abstract]
 OR "emotional awareness"[Title/Abstract]
 OR "emotional expression"[Title/Abstract]
 OR "emotion regulation"[Title/Abstract]
 OR "emotional regulation"[Title/Abstract]
 OR self-control[Title/Abstract]
 OR self-regulation[Title/Abstract]
 OR empathy[Title/Abstract]
 OR prosocial[Title/Abstract]
 OR "social skill"[Title/Abstract]
 OR "social skills"[Title/Abstract]
 OR cooperation[Title/Abstract]
 OR teamwork[Title/Abstract]
 OR responsibility[Title/Abstract]
 OR motivation[Title/Abstract]
 OR enjoyment[Title/Abstract]
 OR autonomy[Title/Abstract]
 OR competence[Title/Abstract]
 OR relatedness[Title/Abstract]
 OR self-efficacy[Title/Abstract]
 OR self-esteem[Title/Abstract]
 OR self-concept[Title/Abstract]
 OR resilience[Title/Abstract]
 OR wellbeing[Title/Abstract]
 OR well-being[Title/Abstract]
 OR anxiety[Title/Abstract]
 OR stress[Title/Abstract]
)
AND
(
 child*[Title/Abstract]
 OR pupil*[Title/Abstract]
 OR "primary school"[Title/Abstract]
 OR "elementary school"[Title/Abstract]
 OR "primary education"[Title/Abstract]
 OR "elementary education"[Title/Abstract]
)
AND
(
 intervention*[Title/Abstract]
 OR program*[Title/Abstract]
 OR programme*[Title/Abstract]
 OR trial[Title/Abstract]
 OR random*[Title/Abstract]
 OR experiment*[Title/Abstract]
 OR quasi-experiment*[Title/Abstract]
 OR controlled[Title/Abstract]
)
AND
("2000/01/01"[Date - Publication] : "2026/05/31"[Date - Publication])

# S3. PRISMA reconciliation

Supplementary Table S3. Final PRISMA counts.

| Stage | Count |
| --- | --- |
| Records identified | 924 |
| Duplicates removed | 80 |
| Other version or erratum removed | 1 |
| Records screened | 843 |
| Records excluded | 712 |
| Reports sought | 131 |
| Reports not retrieved | 12 |
| Reports assessed | 119 |
| Full-text exclusions | 45 |
| Included reports | 74 |
| Independent trial families | 70 |

# **S4. Characteristics of included reports**

The 74 included reports represent 70 trial families. The table summarizes study location, participants, design, intervention and comparison conditions, psychological outcomes, quantitative synthesis category, and companion-report relationships. Full bibliographic metadata, DOI, report identifiers, and effect-level audit fields are provided in the Supplementary Data workbook.

**Supplementary Table S4. Characteristics of included reports.**

| Author (year) | Country/region | Sample and grade/age | Study design | PE intervention and comparator | Psychological outcome domain(s) | Quantitative synthesis category | Trial-family/companion note |
| --- | --- | --- | --- | --- | --- | --- | --- |
| Abdulla et al. (2022) | Maldives | 725 students nested in 30 classes and 4 schools; Fifth grade; age 9–12 years | School-randomized controlled multilevel pre–post study | Eight-hour teacher professional learning plus PE resource support to promote autonomy, competence, and relatedness versus usual PE | Need support/satisfaction/frustration, motivation, self-efficacy, enjoyment, engagement | Broader controlled; Controlled motivation, separate; Context perceptions, separate; Not pooled | Single-report trial family |
| Andrade et al. (2020) | Brazil | 140 elementary children, aged 7–11, Brazil | Cluster-randomized controlled natural experiment | Three 40-min PE classes using exergames versus Three routine curricular PE classes | Mood states; self-esteem | Short-term between-group | Single-report trial family |
| Ang and Penney (2013) | Singapore | Seven Primary 6 PE classes in one primary school | School-based intervention combining quantitative questionnaires with qualitative observations and focus groups | Modified Sport Education unit; no separate quantitative comparator | Confidence, resilience, and social-emotional learning | Narrative only | Single-report trial family |
| Arias-Estero et al. (2020) | Spain | 40 fourth-grade elementary-school students | Short-term pre-post intervention study | TGfU floorball unit; assessments at lessons 1, 10 and 17; no concurrent control | Enjoyment, perceived competence and physical-activity intention | Not pooled | Single-report trial family |
| Berleze and Valentini (2022) | Brazil | 100 randomized (50 mastery-climate, 50 control); Children aged 6–7 from low-income families; all had motor delays, with obesity/overweight or adequate weight. | Randomized controlled trial with nutritional-status strata | Twenty-eight weeks, 56 PE sessions (2 × 90 min/week). Mastery motivational climate with motor-skill training, 15 nutrition-oriented sessions, and six parent-participation sessions versus teacher-centered PE aligned with the school curriculum. | Perceived social acceptance, perceived motor competence, global self-worth. | Broader controlled | Single-report trial family |
| Borowiec et al. (2019) | Poland | 28 deaf children aged 9–13 years | Small randomized controlled trial | Four months of dance lessons with vibrational headphones versus control | Self-esteem | Broader controlled | Single-report trial family |
| Burford et al. (2022) | United States | 402; Elementary school; Grades 2–5 | Acute within-subject repeated-measures condition comparison | Teacher-led versus student-choice HIIT activities within PE over a short assessment period | Enjoyment and perceived competence | Short-term within-child | Single-report trial family |
| Cakir et al. (2025) | Türkiye | Primary-school students in Türkiye | Uncontrolled pre-post study | Physical Activity Cards programme in PE | Enjoyment of physical activity | Uncontrolled pre–post | Single-report trial family |
| Çalık et al. (2018) | Türkiye | 243 children (95 intervention, 148 control), mean age 10.9 years | Controlled pre-post intervention | 14-week Kids’ Athletics programme (3 × 60 min/week) versus no Kids’ Athletics programme | Self-esteem | Narrative only | Single-report trial family |
| Carcelén-Fraile (2025a) | Spain | 120 primary school students, mean age 9.40 ± 0.98, Spain | Randomized controlled trial | Active gamification program based on physical exercise versus Control group | Self-esteem, self-concept, social skills | Not pooled | Linked companion: Carcelén-Fraile (2025b) |
| Carcelén-Fraile et al. (2025b) | Spain | 120 reported participants; eligibility ages 8–11 conflict with reported mean age 6.67 | Nonrandomized controlled intervention study | Active-gamification PE programme versus control | Anxiety and sleep-related outcomes | Not pooled | Linked companion: Carcelén-Fraile (2025a) |
| Castillo-Viera et al. (2021) | Spain | 294 primary-school students | Quasi-experimental pre-post study | Dramatisation-based PE programme | Emotional expressiveness, self-awareness, self-control, motivation, and social skills | Not pooled | Single-report trial family |
| Castle and Howells (2024) | United Kingdom | 97 (50 individualized-target intervention; 47 control); Children aged 6–7 from two primary schools. | Two-session comparative experimental case study | Two timetabled PE lessons. The intervention group received individualized low/mid/high targets in lesson 2; the control repeated the activities without targets. | Enjoyment and perceived competence, plus motor-task performance. | Narrative only | Single-report trial family |
| Chan et al. (2019) | Hong Kong, China | Primary-school children in Hong Kong | Cluster-randomized controlled trial | Assessment-based fundamental movement-skills PE intervention versus usual PE | Perceived physical competence, perceived movement-skill competence, enjoyment, and perceived teacher support (context measure) | Context perceptions, separate; Source-informed controlled | Single-report trial family |
| Chang et al. (2016) | Taiwan, China | 126 sixth-grade students (61 intervention, 65 control) | Class-level quasi-experimental controlled pre-post study | Six weeks of autonomy-supportive PE (2 × 40 min/week) versus teacher-directed PE | Perceived autonomy support, autonomy, and self-determined motivation | Broader controlled; Other constructs, separate; Not pooled | Single-report trial family |
| Chen et al. (2026) | China | 89 second-grade students (44 intervention, 45 control) | Randomized controlled trial | 10-week structured fundamental motor-skills programme (3 sessions/week) versus regular physical activities | Self-regulation, inhibitory control, and emotion regulation | Not pooled | Single-report trial family |
| Cocca et al. (2020) | Mexico | 229 children aged 10–12 after exclusions, Mexico | Quasi-experimental pre–post controlled design | 6-month games-centered PE activities versus Traditional exercise-training PE activities | Psychological wellbeing, self-esteem, stress, anxiety | Broader controlled | Single-report trial family |
| Condello et al. (2021) | Italy | 181 fifth graders, Italy | Class-randomized cross-over trial | Six-month multisport enriched PE intervention centered on team games versus Traditional PE | Prosocial behavior (empathy/cooperation) and antisocial behavior (quick-temperedness) | Broader controlled; Source-informed controlled | Single-report trial family |
| Dallolio et al. (2016) | Italy | Primary-school pupils participating in an enhanced PE programme | Nonrandomized cluster-controlled study with adjusted analysis | Enhanced PE programme versus standard PE | Perceived competence and related psychosocial indicators | Broader controlled | Single-report trial family |
| Duncan et al. (2018) | United Kingdom | 94 children aged 6–7 years (53 intervention, 41 control) | Cluster-randomized controlled pre-post study | 10-week integrated neuromuscular training replacing one weekly PE lesson versus usual PE | Physical self-efficacy | Not pooled | Single-report trial family |
| Eckert et al. (2026) | Denmark | 1,357 randomized; 1,175 consented; Primary school; age 7–11 years | Three-arm cluster-randomized controlled trial | Four-month FF10 multisport PE at approximately 1.5 or 3 sessions/week versus standard PE | KIDSCREEN psychosocial domains, social support/peers, school environment, global and physical self-worth | Broader controlled; Physical-health outcomes, separate | Single-report trial family |
| Fairclough et al. (2016) | United Kingdom | 139 children aged 10–11 years from four schools | Non-equivalent cluster pilot trial | Six-week Born to Move programme plus regular PE versus regular PE | Enjoyment and perceived competence | Broader controlled | Single-report trial family |
| Fairclough et al. (2024) | United Kingdom | Primary-school children participating in a feasibility study | Uncontrolled pre-post feasibility study | 10-week Move Well, Feel Good motor-competence intervention | Mental-health difficulties, prosocial behaviour, peer support, self-worth, and wellbeing | Overlapping components; Uncontrolled pre–post | Single-report trial family |
| Folleto et al. (2016) | Brazil | 16 enrolled children aged 6–8 years; first grade | Uncontrolled pre–post, mixed methods | Yoga in PE, 12 weeks, two 45-minute lessons/week; no concurrent control | Pictorial cognitive/motor competence and social/maternal acceptance; separate qualitative interviews | Not pooled | Single-report trial family |
| Fuller et al. (2017) | Denmark | 546 total; 482 with complete well-being data; Fifth grade; age 10–12 years | School-block-randomized controlled two-cohort study | Eleven weeks; two 45-min PE periods/week combining football and health education versus regular PE | Physical, emotional, social, and school-related well-being | Broader controlled; Physical-health outcomes, separate | Single-report trial family |
| Gaspar et al. (2021) | Spain | 111 (54 intervention; 57 control); Fifth and sixth grade; mean age 10.95 years | Class-randomized quasi-experimental controlled pre–post study | Sixteen curricular PE sessions; TGfU with questioning versus the same small-sided-game unit without questioning | Autonomy, competence, relatedness, autonomous/controlled motivation, amotivation, perceived ability, PA intention | Other constructs, separate; Not pooled | Single-report trial family |
| Gentile et al. (2020) | Italy, Germany, and Lithuania | 342 reported; Table 1 totals 331 assigned; Schoolchildren; ESA designed for age 7–14 years | Controlled pre–post study; allocation method incompletely reported | Fourteen weeks, 27 cognitively enriched sport/PE units plus four parent seminars versus ordinary PE | Intrinsic, identified, introjected, external motivation, amotivation, family and peer social support | Not pooled | Single-report trial family |
| Gil-Madrona et al. (2019) | Spain | 204 fifth- and sixth-grade students from seven schools | Quasi-experimental pre-post intervention study | 20-session Delfos appropriate-behaviour programme in PE | Appropriate behaviour, fair play, and social skills | Not pooled | Single-report trial family |
| Goñi and Zulaika (2000) | Spain | 75 (46 intervention; 29 control); Fifth grade; age 10–11 years | Controlled non-randomized pre–post study | 48 curricular PE lessons over 6 months; individualized goals, participatory/noncompetitive games, and praise versus usual PE | General self-concept, nonacademic self-concept, physical self-worth, global self-esteem, physical self-perceptions | Broader controlled; Overlapping components | Single-report trial family |
| Harvey et al. (2017) | United States | 94 elementary pupils (of 173 total); Elementary subgroup: Grades 4–5; elementary results are reported separately from middle school. | One-group quasi-experimental pretest–posttest study | A 33-lesson Tactical Games Model basketball unit delivered in curricular PE; no concurrent comparison group. | Autonomy, competence, relatedness, intrinsic/identified/introjected/external motivation, amotivation. | Other constructs, separate; Uncontrolled pre–post | Single-report trial family |
| Huéscar Hernández et al. (2020) | Spain | 102 (49 intervention; 53 control); Fifth and sixth grade; age 10–13 years | Quasi-experimental controlled pre–post study | Nine months of curricular PE using autonomy support, dialogic learning, interactive groups, and family volunteers versus the same units without the intervention | Autonomy, competence, relatedness, self-determined motivation in PE, PA intention and behavior | Not pooled | Single-report trial family |
| Invernizzi et al. (2022) | Italy | 183 primary-school children allocated to two intervention groups and a control group | Class-randomized controlled trial | 12-week multiteaching psychomotor programme versus conventional PE | Perceived physical ability (PSES) and enjoyment (PACES) | Broader controlled | Single-report trial family |
| Jimenez-Garcia et al. (2024) | Colombia | Children aged 8–12 years | Cluster-controlled feasibility study with mixed-model analysis | Neuromuscular warm-up based on physical literacy versus regular warm-up/PE | Motivation, confidence, and perceived competence | Source-informed controlled | Single-report trial family |
| Kliziene et al. (2021) | Lithuania | Primary school children aged 6–7 and 8–9, Lithuania | Intervention study with experimental and control groups | Eight-month purposefully constructed PE program versus Control group in same school context | Emotional well-being: somatic anxiety, personality anxiety, social anxiety | Not pooled | Single-report trial family |
| Kolovelonis et al. (2013) | Greece | 120 fifth- and sixth-grade elementary PE students | Randomized five-condition acute learning experiment | Self-regulated-learning conditions during basketball dribbling practice | Motivational beliefs and self-regulated performance calibration | Short-term between-group | Single-report trial family |
| Kuspratiwi et al. (2026) | Indonesia | 192 Grade 4–5 students, aged 9–10, Indonesia | Cluster-randomized controlled trial | 12-week cooperative circuit games; weekly 55-minute sessions versus regular PE instruction | SDQ: prosocial behavior; total difficulties / social-emotional functioning | Broader controlled | Single-report trial family |
| Lakes and Hoyt (2004) | United States | 207 children from kindergarten through Grade 5 | Class-randomized controlled trial | Three-month school-based martial arts programme versus traditional PE | Affective and cognitive self-regulation, prosocial behaviour, classroom conduct | Narrative only | Single-report trial family |
| Likesas and Zachopoulou (2006) | Greece | 232 (135 intervention; 97 control); Elementary grades 1–6; age 6–11 years | Class-level controlled pre–post study with partial random assignment | 22 curricular dance lessons, 2 × 45 min/week, November–April; music-and-movement/creative model versus teacher-centered dance instruction | Effort-interest and pleasure/intrinsic motivation | Broader controlled | Single-report trial family |
| Liu et al. (2025) | China | Rural primary-school children in Nanxian County, Hunan Province | Cluster-randomized controlled trial | Team-building sports games versus usual school provision | Resilience: affect control, positive thinking, and help-seeking | Not pooled | Single-report trial family |
| Luna et al. (2020) | Spain | 146 children aged 10–12, Spain | Quasi-experimental pre–post controlled design; cluster randomized assignment | Sport Education Model intervention; 18 sessions of 50 min versus Traditional direct-instruction intervention | Positive affect, negative affect, anxiety, depression, social stress | Not pooled | Linked companion: Luna (2021) |
| Luna et al. (2021) | Spain | 148 reported students, ages 10–13; outcome-specific arm denominators unresolved | Repeated pre–post comparison with active control; outcome-specific allocation/variance limits | MooN program based on the Sport Education Model versus Traditional model of direct instruction active control | Perceived interpersonal/intrapersonal capability (IAMI-M40 confidence ratings) | Not pooled | Linked companion: Luna (2020) |
| Luptáková et al. (2025) | Slovakia | 266 primary-school students in grades 1–4, Slovakia | Class-configured comparative pre–post | Five teaching configurations: PET, GT+PET, PET+AT, GT+C and GT+AT; no common untreated comparator | Self-reported physical literacy and basic motor competencies | Not pooled | Single-report trial family |
| Lwin and Malik (2014) | Singapore | 398 analyzed (454 initially); Fifth grade; mean age 10.2 years | Class-randomized 2 × 2 factorial controlled study | One weekly PE lesson with Wii exergames for 6 weeks versus regular PE, crossed with threat- or coping-framed health messages | Physical-activity attitude, self-efficacy, perceived behavioral control, social norms, intention | Not pooled | Single-report trial family |
| Madsen et al. (2020) | Denmark | 3,061 randomized (2,533 intervention; 528 control), as reported in Madsen (2020); fifth grade, age 10–12 years. The companion report gives a different parent-sample total (see Ryom row). | Large-scale school cluster-randomized controlled trial | Eleven weeks, 2 × 45 min/week football and health education; one session often replaced PE and the second replaced another subject | Physical, psychological, peer/social-support, and school well-being | Broader controlled; Physical-health outcomes, separate | Linked companion: Ryom (2022) |
| Mayorga-Vega et al. (2012) | Spain | 75 sixth-grade pupils (69 analysed), mean age 11.1 years | Cluster-randomized controlled trial | Eight-week physical-fitness circuit programme during PE versus usual PE | Physical self-concept and self-esteem | Broader controlled; Physical-health outcomes, separate | Single-report trial family |
| Miller et al. (2015) | Australia | 168 Year 6 students (97 intervention, 71 wait-list control); mean age 11.2 years | Cluster-randomized controlled trial | Games-based professional-learning programme for PE teachers, implemented in primary-school PE, versus wait-list/usual practice | Athletic competence and related psychosocial outcomes | Source-informed controlled | Single-report trial family |
| Mira et al. (2026) | Spain | 97 enrolled (41/56); 94 paired observations (38/56); Table 2 cell-specific denominator unidentified | Non-randomized pre–post control-group mixed-methods design | Eight 45-min sessions over three weeks combining cooperative games, yoga-based relaxation, and motor activities versus a control group | Children’s Emotional Adjustment Scale: source-defined domains, without conflating them with ability tests | Not pooled | Single-report trial family |
| Neira-Navarrete et al. (2024) | Chile | 40 reported participants; one fifth-grade class; mean age 11.47 years; reported sex counts do not reconcile | Uncontrolled pre–post | Cooperative games and reflection, 12 weeks, one 60-minute session/week in addition to PE; no concurrent control | Self-perceived fitness (IFIS) and motor competence (SEMOK), distinct from motor performance | Not pooled; Overlapping components; Uncontrolled pre–post | Single-report trial family |
| Nigg (2024) | Hawai‘i, USA (education/ethics jurisdiction) | 157 consented students across K–6; questionnaires restricted to grades 4–6; analyzed survey arm sizes not reported | Grades 4–6 classes randomly allocated (4 intervention/2 control); younger classes intervention only | Twelve-week Hatha yoga programme (20–30 min/week) versus regular PE | Psychosocial indicators and physical-activity outcomes | Other constructs, separate; Not pooled | Single-report trial family |
| Park et al. (2017) | South Korea | 48 analyzed children: multicultural arms 12/13 and single-cultural arms 11/12; abstract reports a conflicting total | Small nonrandomized controlled study | Regular PE programme versus comparison condition | Sociality and psychological problems | Broader controlled | Single-report trial family |
| Pesce et al. (2021) | Italy | 156 third- and fourth-graders, aged 8–9, Italy | Class-randomized trial | Two-year theory-based PE intervention designed to train self-control versus Traditional/regular PE | Quick-temperedness, disruptiveness, hot executive function, self-control | Source-informed controlled | Single-report trial family |
| Pinto-Escalona et al. (2024) | France, Germany, Poland, Portugal, and Spain | Primary-school children from five European countries in a multicountry school sample | Multicountry cluster-randomized controlled trial | One-year school-based karate programme versus usual PE | Psychosocial functioning, emotional symptoms, peer problems, and prosocial behaviour | Not pooled | Single-report trial family |
| Pippi et al. (2020) | Italy | 702; First year of primary school; age 6 years | Uncontrolled pre–post pilot study | Eight months; two specialist-led curricular PE hours/week plus four nutrition-education meetings; no control group | Physical, emotional, self-esteem, family, friends, and school well-being domains | Overlapping components; Physical-health outcomes, separate; Uncontrolled pre–post | Single-report trial family |
| Pope et al. (2015) | United States | Children participating in an after-school/school-based exergaming programme | Uncontrolled pre-post study | Dance-based exergaming programme | Physical-activity self-efficacy and stage of change | Not pooled | Single-report trial family |
| Pozo Rosado et al. (2022) | Spain | Primary-school students participating in a TPSR PE programme | Nonrandomized controlled pre-post study | Teaching Personal and Social Responsibility programme versus usual PE | Empathy, emotional awareness, and emotional intelligence | Broader controlled; Overlapping components | Single-report trial family |
| Rice et al. (2025) | United Kingdom | 63 Year 5 pupils aged 9–10 years from three primary schools | Single-group pre-post feasibility study | 10-week gamified PE unit (one 60-min lesson/week) | Emotional intelligence and motivational/need-related outcomes | Narrative only | Single-report trial family |
| Rigon et al. (2024) | Italy | Second- and fourth-grade primary-school students in Italy | Class-randomized controlled trial | Rhythmic “thinking system” PE approach versus conventional PE | Enjoyment (PACES), grade-2 pictorial motor competence (PMSC-2) and grade-4 perceived physical ability (PSES) | Not pooled | Single-report trial family |
| Rivera-Pérez et al. (2021) | Spain | 40 primary-school students aged 10–12 years | One-group pre-post study | Eight-week cooperative-learning PE programme (16 sessions) | Emotional intelligence and achievement-goal orientations | Narrative only | Single-report trial family |
| Rodríguez-Negro and Yanci (2022) | Spain | 168 primary-school children aged 8–12 years | Two-group pre-post intervention study | Eight-week tactical games model versus direct instructional model in PE | Impulse control | Not pooled | Single-report trial family |
| Ruiz-Montero et al. (2025) | Spain | Spanish primary fifth/sixth-grade students; final analyzed SL n=35 and traditional n=38; age about 10–11 years | Quasi-experimental longitudinal pre-post with comparison group | 10-week intergenerational service-learning through physical activities | Empathy; respect; sociability; leadership; PE importance | Not pooled | Single-report trial family |
| Russo et al. (2025) | Italy | 64 students aged 9–10 years | Mixed-methods intervention study | Seven-month PE intervention integrating respect, fair play, and collaboration; comparison condition as reported | Respect, collaboration, self-esteem, and fair-play decision making | Narrative only | Single-report trial family |
| Ryom et al. (2022) | Denmark | 1,122 ethnic-minority children aged 10–12 years. This companion report gives parent-sample n=3,062, whereas Madsen (2020) reports n=3,061; the source difference is retained. | Secondary subgroup report from the same cluster RCT as Madsen et al. (2020) | Same 11-week '11 for Health in Denmark' program and control condition | School/learning well-being, health knowledge, fitness | Narrative only | Linked companion: Madsen (2020) |
| Sindiani et al. (2025) | Israel | 260 students in Grades 4–6 from two schools (8 intervention classes, 3 control classes) | Convergent mixed-method controlled intervention study | Three 45-min SEL-modified PE sessions versus a standard 45-min PE class | Teamwork, self-awareness, creative thinking, and reflection | Narrative only | Single-report trial family |
| Sit et al. (2019) | Hong Kong, China | Children with developmental coordination disorder in Hong Kong primary schools | Randomized controlled trial | School-based physical-activity intervention versus control | Enjoyment and psychosocial outcomes alongside motor outcomes | Not pooled | Single-report trial family |
| Sotos-Martínez et al. (2023) | Spain | 72 (35 gamified; 37 control); Elementary school; age 9–11 years | Cluster-randomized natural experiment / controlled pre–post study | Ten gamified PE sessions using ClassDojo and PBL/MDA models versus traditional PE | Intrinsic motivation, identified, introjected, external regulation, amotivation | Broader controlled; Other constructs, separate | Single-report trial family |
| Theodorakou and Zervas (2003) | Greece | 107 fifth- and sixth-grade children, aged 11–12, Greece | Pre–post controlled intervention | Creative movement teaching method for 3 months / 24 teaching hours versus Traditional teaching method | SPPC scholastic/athletic competence, social acceptance, physical appearance, behavioral conduct and global self-worth | Not pooled | Single-report trial family |
| van Rossum et al. (2025) | England, United Kingdom | 229 children from nine primary schools (128 intervention, 101 wait-list control); mean age 9.1 years | Cluster-randomized controlled trial with adjusted analysis | Nine-week MOGBA complex movement-skills intervention versus wait-list/usual PE | Physical self-perception, game perception, and game perception relative to others | Source-informed controlled | Single-report trial family |
| Vanaclocha-Amat et al. (2026) | Spain | 254; Primary school; mean age 11.10 years | Three-arm cluster-randomized controlled trial | Eight-week FunctionalHIIT during PE, with or without gamification, versus usual PE/control | Enjoyment, intrinsic motivation, identified regulation, amotivation | Broader controlled | Single-report trial family |
| Vasilopoulos and Dumontheil (2026) | United Kingdom | 104 children aged 6–7 years | Three-arm class-randomized pilot trial | Creative dance and yoga versus usual PE; shared control retained within one trial family | Behavioral self-regulation, task self-control, creativity-related behavioral strengths and companion mental-health outcomes | Broader controlled; Other constructs, separate; Not pooled | Single-report trial family |
| Xiang et al. (2006) | United States | 113 complete cases (66 boys, 47 girls); Fourth-grade pupils in one Texas elementary school; participants had taken part in Roadrunners since kindergarten. | Uncontrolled longitudinal pre–post program evaluation | Roadrunners was integrated into regular PE. Pupils attended daily 30-min PE and completed a weekly run/walk across one school year; there was no concurrent comparison group. | Achievement goals, expectancy beliefs, task values, intention for future running participation, persistence/effort. | Other constructs, separate; Uncontrolled pre–post | Single-report trial family |
| Xie et al. (2025) | China | 130 primary-school students, mean age 10.9 years | Quasi-experimental controlled pre-post study | 12-week TPSR-based basketball PE programme versus usual PE | Personal and social responsibility and prosocial behaviour | Narrative only | Single-report trial family |
| Xu et al. (2025) | China | 203 children aged 8–10 years | Cluster-randomized controlled trial | 10-week cognitively engaging physical-activity programme (3 × 45 min/week) versus traditional PE | Self-control and its dimensions | Overlapping components; Source-informed controlled | Linked companion: Xu (2026) |
| Xu et al. (2026) | China | School-age children participating in a two-cluster randomized trial | Two-cluster randomized controlled trial | 11-week cognitively engaging PE versus traditional PE | Overall mental health and resilience | Not pooled | Linked companion: Xu (2025) |
| Yan et al. (2024) | China | 715 consenting pupils in 20 schools; 591 provided valid questionnaires at both assessments. Table 5 arm headers total 733 (source discrepancy; see S11). Grades 4–6; age 10–13 years. | School cluster-randomized controlled trial | MASTER teacher-education and game-based basketball PE versus usual teaching practice | Athletic competence, enjoyment, well-being, autonomous and intrinsic motivation, and introjected/external regulation | Controlled motivation, separate; Source-informed controlled | Single-report trial family |

# S5. Methodological appraisal

All 74 reports were represented in the item-level Mixed Methods Appraisal Tool 2018 ledger (MMAT; Hong et al., 2018). Each report received two screening questions and five design-specific criteria; nine mixed-methods reports additionally received qualitative and integration criteria, yielding 608 item rows. Judgments were Yes, No, or Can’t tell, with source locations and reasons. No total score, percentage ranking, or quality threshold determined model eligibility. During revision, targeted source checks corrected ten item records, including a random-number cognitive task mistakenly cited as evidence of random allocation, an unrelated thesis chapter cited for recruitment, and teacher retention mistakenly used to support student outcome completeness. Remaining historical judgments and newly checked records are distinguished in the ledger; the targeted checks are not presented as a new independent appraisal of every item.

Supplementary Table S5. MMAT item inventory.

| MMAT component | Yes | No | Can’t tell | Items |
| --- | --- | --- | --- | --- |
| Mixed methods integration | 26 | 0 | 19 | 45 |
| Qualitative research component | 36 | 0 | 9 | 45 |
| Quantitative non-randomized study | 44 | 15 | 136 | 195 |
| Quantitative randomized controlled trial | 35 | 2 | 138 | 175 |
| Screening questions | 141 | 0 | 7 | 148 |

# S6. Effect sizes, dependence, and model estimates

Controlled effects were expressed as Hedges’ g with a small-sample correction (Hedges, 1981). For raw posttest data, the intervention-minus-control mean difference was divided by the pooled within-group standard deviation, then multiplied by J, approximated by 1 − 3/[4(nE + nC) − 9]. The variance approximation was 1/nE + 1/nC + g²/[2(nE + nC − 2)], with effective sample sizes substituted only in the declared design-effect scenarios. Raw posttest comparisons do not become baseline-adjusted change effects merely because both groups also had pretests. Controlled change and posttest estimands require design-specific interpretation (Morris, 2008).

For adjusted raw coefficients, the coefficient and its standard error were divided by an identified standardizer; when only a 95% interval was available, the standard error was approximated by its width divided by 3.92. For reported standardized coefficients and intervals, J was applied to both the estimate and its standard error. Yan and Miller required an additional conditional approximation: a scale factor inferred from the reported standardized effect and adjusted raw contrast. This treats the denominator as fixed and requires both quantities to correspond to the same contrast. A declared sensitivity omitted these reports. The source-informed set is therefore not assumption-free. Chan’s narrative standard error was preferred over a conflicting table interval, and a separate sensitivity omitted the whole report.

Uncontrolled effects standardized within-group change and did not estimate an intervention-versus-control effect (Morris and DeShon, 2002). Their sampling variances depended on the pre–post correlation, which was varied separately from the working correlation among effect estimates. Source formulas, reported versus derived quantities, and each assumption are given in the Analysis_Routing worksheet. Source-level reporting contradictions were retained as uncertainty, not silently resolved by choosing a favorable value.

Models used rma.mv in metafor (Viechtbauer, 2010) and CR2 covariance estimation with Satterthwaite degrees of freedom (Tipton, 2015; Pustejovsky and Tipton, 2018). We report effect and family counts, estimates, CR2 standard errors, confidence intervals, degrees of freedom and p values, and both variance components. Approximate multilevel I² divides total estimated heterogeneity by that heterogeneity plus the mean diagonal sampling variance; it is a descriptive, working-correlation-dependent summary, not an absolute effect-size range (Borenstein et al., 2017). Prediction intervals come from the fitted multilevel model using containment-based t inference in predict.rma.mv. They are not CR2 prediction intervals. They describe a model-implied distribution of effects in comparable contexts and are particularly unstable with three or five families (Higgins et al., 2009; IntHout et al., 2016).

Supplementary Table S6. Final model estimates.

| Analysis | Effects / families | g [95% CR2 CI] | SE / df | Raw p | Holm p | Model PI |
| --- | --- | --- | --- | --- | --- | --- |
| Core source-informed | 5 / 3 | 0.947 [-0.774, 2.667] | 0.399 / 2.00 | = .141 | = .424 | [-2.387, 4.280] |
| Adjacent source-informed | 13 / 5 | 0.215 [-0.386, 0.817] | 0.205 / 3.51 | = .360 | = .424 | [-1.368, 1.799] |
| Combined source-informed | 18 / 8 | 0.421 [-0.001, 0.843] | 0.175 / 6.35 | = .0504 | Not applied | [-0.984, 1.826] |
| Broader controlled | 126 / 28 | 0.381 [0.135, 0.627] | 0.120 / 26.73 | = .004 | Not applied | [-0.913, 1.676] |
| Broader core | 15 / 7 | 1.024 [-0.074, 2.122] | 0.447 / 5.93 | = .063 | Not applied | [-2.015, 4.062] |
| Broader adjacent | 111 / 24 | 0.359 [0.102, 0.615] | 0.124 / 22.69 | = .008 | Not applied | [-0.886, 1.603] |
| Uncontrolled change | 30 / 6 | 0.128 [-0.377, 0.632] | 0.196 / 4.99 | = .544 | Not applied | [-1.220, 1.475] |

# S7. Declared exploratory sensitivity analyses

Table S7 summarizes the declared sensitivity analyses. Full moderator coefficients, CR2 inference, raw and adjusted p values, and the corresponding multiplicity families are reported in the Model_Results_R1 worksheet of the Supplementary Data workbook; the adjustment procedures are described in section S10.

Supplementary Table S7. Declared exploratory sensitivity analyses.

| Exploratory analysis | Effects / families | Estimate [95% CR2 CI] | p |
| --- | --- | --- | --- |
| Core, working correlation .30 | 5 / 3 | 0.953 [-0.738, 2.645] | = .136 |
| Core, working correlation .80 | 5 / 3 | 0.947 [-0.775, 2.668] | = .142 |
| Broader, external ICC .01 | 126 / 28 | 0.384 [0.138, 0.630] | = .004 |
| Broader, external ICC .10 | 126 / 28 | 0.379 [0.134, 0.625] | = .004 |
| Core, all components instead of total | 8 / 3 | 0.850 [-0.158, 1.858] | = .067 |
| Alternative taxonomy, agency/core | 11 / 8 | 0.437 [-0.129, 1.002] | = .111 |
| Alternative taxonomy, adjacent | 7 / 3 | 0.385 [-0.983, 1.753] | = .270 |
| Combined, without inferred standardizers | 12 / 6 | 0.452 [-0.248, 1.153] | = .158 |
| Combined, without Chan report | 15 / 7 | 0.505 [0.064, 0.947] | = .032 |
| Broader, source-ambiguity omission | 95 / 25 | 0.338 [0.142, 0.534] | = .002 |
| Uncontrolled, without Neira report | 24 / 5 | 0.138 [-0.532, 0.807] | = .599 |

# S8. Eligible results not included in quantitative synthesis

89 candidate outcome records from 31 reports and 29 families were not pooled because of incompatible or inadequately identified numerical/design information. Other measures were separated for construct scope, overlapping representation or short-term design, rather than equated with missing variances. The data workbook records both types of decisions and their sources.

Supplementary Table S8 summarizes remaining unpooled results by report. Full row-level values, source locators, reasons, and old-to-new transitions are in the Supplementary Data workbook.

Supplementary Table S8. Eligible effects not included in quantitative synthesis.

| Report | Outcomes | Records | Source-specific reason |
| --- | --- | --- | --- |
| Abdulla et al. (2022) | Teacher-rated behavioral engagement | 1 | Arm-weighted and reported overall means disagree, and the overall baseline SD is incompatible with arm SDs; the correct cells are unidentified. |
| Arias-Estero et al. (2020) | Psychosocial outcomes | 1 | Only medians/nonparametric summaries are available; no unsupported distributional conversion is made. |
| Carcelén-Fraile (2025a) | Self-esteem; Impulsiveness | 2 | Reproducible numerical contradictions affect the outcome scores and baseline comparison; a reliable standardized contrast could not be recovered. |
| Carcelén-Fraile et al. (2025b) | SCAS-C-8 total anxiety | 1 | Mean age conflicts with the stated range; table n conflicts with F degrees of freedom; several narrative d values conflict with table changes. |
| Castillo Viera et al. (2021) | Emotional expressiveness; Self-control; Motivation; Self-awareness; Social skills | 5 | Sampling of classes is not treatment randomization. The treatment assignment hierarchy and class counts are absent, and no clustered analysis was used. |
| Chang et al. (2016) | Perceived autonomy in PE | 1 | The retained autonomy effect construction could not be reconciled with compatible source estimates and uncertainty; other recoverable outcomes are analyzed separately. |
| Chen et al. (2026) | Self-regulation; Emotion regulation | 2 | Excluded from quantitative synthesis because source reports conflict; Scale description says higher scores indicate poorer function, yet the intervention-group score increased and is interpreted as improvement. Reported posttest d = +0.758. Excluded from quantitative synthesis because source reports conflict; Table reports nonsignificant interaction F = 3.408, whereas abstract states F = 4.681 and narrative reports significant posttest d = -0.459, p = .033. |
| Duncan et al. (2018) | Physical self-efficacy | 1 | Excluded from quantitative synthesis because a required cell N/covariance/convertible statistic is unavailable; Effect was primarily among boys; do not use a single unstratified effect without source data. |
| Folleto et al. (2016) | Perceived competence/social acceptance | 1 | Full text is eligible, but no compatible mean/SD, adjusted estimate with SE, or convertible statistic is reported. |
| Gaspar et al. (2021) | Autonomy; Competence; Relatedness; Autonomous motivation; Amotivation; Perceptions of ability | 6 | Sex-specific arm sizes are unavailable. The reported sex-stratified means and SDs cannot be combined into a weighted overall mean, SD and sampling variance. |
| Gentile et al. (2020) | Intrinsic motivation; Identified regulation; Introjected regulation; External regulation; Amotivation | 5 | The phrase that classes were split does not establish individual randomization; no cluster count or cluster-aware uncertainty can be reconstructed. |
| Gil-Madrona et al. (2019) | Appropriate behavior when winning | 1 | Excluded from quantitative synthesis because source reports conflict; Article reports lower posttest scores as improvement, which conflicts with the construct label. |
| Huéscar Hernández et al. (2020) | Autonomy need satisfaction; Competence need satisfaction; Relatedness need satisfaction; Self-determined motivation in PE | 4 | The treatment-assignment hierarchy and class counts are unspecified in a two-school sample analyzed at pupil level; allocation-aware uncertainty is unidentified. |
| Kliziene et al. (2021) | Personality anxiety - Grade 1; Social anxiety - Grade 1; Personality anxiety - Grade 2; Social anxiety - Grade 2 | 4 | The report simultaneously describes four schools and says all children attended the same school; class counts and arm allocation are absent, so a valid cluster correction cannot be reconstructed. |
| Liu et al. (2025) | Affect control; Positive thinking; Help-seeking | 3 | One cluster per arm makes treatment perfectly confounded with class; no statistical correction can recover an intervention effect from this design. |
| Luna et al. (2020) | Positive affect; Negative affect; Anxiety; Depression; Social stress | 5 | Outcome totals differ across measures, analyzed arm sizes are unidentified, and F/p pairs are inconsistent. Enrolled arm sizes cannot substitute for endpoint denominators. |
| Luna et al. (2021) | Interpersonal socio-emotional self-efficacy; Intrapersonal socio-emotional self-efficacy | 2 | The IAMI-M40 confidence measure concerns perceived capability. Source-level allocation and variance limitations prevent a compatible pooled effect; the construct label alone is not the reason for exclusion. |
| Luptáková et al. (2025) | Psychosocial/physical-literacy outcomes | 1 | Nonparametric summaries cannot be pooled as Hedges g without unsupported assumptions. |
| Lwin & Malik (2014) | Physical-activity self-efficacy; Perceived behavioral control | 2 | The PE intervention is crossed with message framing; adjusted results do not provide a compatible raw standardized contrast and sampling variance. |
| Mira et al. (2026) | Mood repair; Anxiety control; Temperament control; Social assertiveness | 4 | Enrolled intervention n = 41 differs from paired n = 38; the denominator underlying Table 2 is unidentified. One intact group per condition also confounds treatment and group. Historical identifiers refer to this same report. |
| Neira-Navarrete et al. (2024) | Perceived object-control competence (SEMOK) | 1 | The object-movement post mean (8.17) exceeds the five-point scale and conflicts with the reported overall mean (4.15); no corrected value is established. |
| Nigg (2024) | Physical-activity self-efficacy; Perceived stress; PA attitude | 3 | Analyzed survey arm sizes are not reported; enrollment covers grades not surveyed, and the stress F degrees of freedom do not identify the required arm denominators. |
| Pinto-Escalona et al. (2024) | Psychosocial difficulties; Emotional symptoms; Peer problems; Prosocial behavior | 4 | The formal-version supplement labels Table 4 means/SDs as adjusted, whereas raw Table 5 cells lack cell-specific sample sizes. Compatible allocation-aware uncertainty is unavailable. |
| Pope et al. (2015) | Exercise self-efficacy | 1 | The study reports 212 recruited participants and 205 in stage-of-change strata, but the psychological endpoint denominator cannot be uniquely established. |
| Rigon et al. (2024) | PE enjoyment (2nd elementary); Perceived movement-skill competence (2nd elementary); PE enjoyment (4th elementary); Physical self-efficacy (4th elementary) | 4 | For each effect, treatment is perfectly confounded with the single section A/B class. |
| Rodríguez-Negro and Yanci (2022) | Impulse control | 1 | The phrase 'divided into two groups and each group was randomly assigned' does not establish individual randomization, class count, or independent observations. |
| Ruiz-Montero et al. (2025) | Empathy; Respect; Sociability; Leadership; PEIQ importance; PEIQ relative value; PEIQ usefulness | 7 | Treatment is perfectly associated with grade. PE-importance items also lack a compatible composite SD; a cluster adjustment cannot repair grade-treatment confounding. |
| Sit et al. (2019) | Enjoyment | 1 | The companion report does not provide a compatible intervention contrast with sampling variance. |
| Theodorakou and Zervas (2003) | Global self-worth - boys; Global self-worth - girls; Behavioral conduct - boys; Behavioral conduct - girls; Scholastic competence; Social acceptance; Athletic competence; Physical appearance; Scholastic competence; Social acceptance; Athletic competence; Physical appearance | 12 | Allocation-aware uncertainty is unidentified. Behavioral conduct is a self-perception, not demonstrated skill. A duplicated appearance cell and the sex-specific scoring structure are preserved as source uncertainties. |
| Vasilopoulos and Dumontheil (2026) | Self-control | 1 | The retained delay-of-gratification outcome is binary; its reported t-derived g and generic SMD variance do not establish a compatible continuous-effect estimand. |
| Xu et al. (2026) | General mental health; Resilience | 2 | Two-class trial with one class per arm; treatment is inseparable from class. |

Note. All reports listed in this table remained included in the systematic review; only the affected results were excluded from quantitative synthesis.

# S9. Reasons for non-pooling

Supplementary Table S9. Reasons for non-pooling.

| Reason summary | Records | Families |
| --- | --- | --- |
| Adjusted factorial estimates without compatible standardization / covariance | 2 | 1 |
| Binary outcome incompatible with the retained continuous-effect calculation | 1 | 1 |
| Contradictory source scores or statistical reporting | 8 | 5 |
| Missing or incompatible analyzed denominators / sampling uncertainty | 21 | 7 |
| Original outcome-specific source unavailable | 1 | 1 |
| Rank-based summaries without a compatible standardized mean contrast | 3 | 3 |
| Unidentified allocation or treatment–cluster confounding | 53 | 12 |

Reasons summarize the primary obstacle for each retained unpooled record; categories do not rank overall study quality. Related family counts may overlap.

# S10. Reproducibility and data availability

During revision, OpenAI ChatGPT and Codex assisted with computational and source checks, including code preparation, consistency checks and source-linked record reconciliation. These checks do not constitute independent duplicate human review. Source-linked amendments and unresolved issues are documented in the Supplementary Data workbook, and the reproducibility archive contains the executed analysis scripts and outputs. The authors remain responsible for the review decisions, numerical results and interpretation.

Allocation and analysis units, arm sizes, cluster counts, and available ICC information determined quantitative routing. External-ICC scenarios used design effects of 1 + (m − 1)ICC, with .01, .05, and .10. School allocation was not corrected using a classroom ICC as though the two levels were equivalent. Recoverable raw contrasts with two allocation clusters per arm could enter the broader sensitivity set, consistently across reports; a single cluster per condition remained unsuitable because treatment and cluster were confounded. Unequal cluster sizes, sparse clusters, and nonrandom allocation remain limitations of these approximations. Uncontrolled pre–post correlations were varied over .20, .50, and .80, retaining the one reported correlation where available.

Additional revision analyses separated broader core and adjacent outcomes, replaced Xu’s total by all four components, and used one alternative taxonomy that placed perceived competence/self-beliefs within a broader self-awareness or agency category. These were specified from measurement and theory rather than chosen by significance. The alternative taxonomy is a stress test of an operational distinction, not evidence that one taxonomy is uniquely correct. Further source-ambiguity sensitivities omitted Eckert, Fuller and Likesas from the broader set, and Neira-Navarrete from uncontrolled change. The former addresses uncertainty about the school-level model, arm denominators and score description, respectively; the latter addresses a contradictory source component cell. These omissions were source-based, not selected by effect magnitude.

Holm adjustment covered three main tests: the core mean, adjacent mean, and adjacent-minus-core contrast (Holm, 1979). This is a multiple-testing family, not a claim of prospective confirmation. Domain coefficients and the two continuous moderators formed separate exploratory BH-FDR families (Benjamini and Hochberg, 1995). Duration and pedagogical features were not entered into an incomplete moderator search. Leave-one-family-out analyses retained all families in the main model and were interpreted as influence descriptions, not automatic deletion rules (Viechtbauer and Cheung, 2010).

Routine funnel-asymmetry tests were not considered reliable for the three- and five-family primary sets. For the broader evidence, an exploratory analysis formed one generalized least-squares composite per family using the declared working covariance, then regressed composites on the square root of 1/nE + 1/nC with random-effects REML and Knapp–Hartung inference. Only families with one consistent, finite pair of arm sample sizes were eligible; multiarm or outcome-varying counts and unresolved analyzed arm sizes were not silently combined. This avoids treating 126 correlated rows as independent trials and avoids directly regressing SMDs on their potentially coupled standard errors (Pustejovsky and Rodgers, 2019). It remains a sample-size association in a heterogeneous selected subset, not a definitive test or adjustment for publication bias. Statistical interpretation used two-sided p values and 95% intervals, with Holm-adjusted p < .05 for the stated three-test family. No common minimally important difference was established across these heterogeneous scales; a generic standardized-effect cutoff was not used to label practical benefit. GLS composites can assign negative weights to individual outcomes. We therefore retained the weights and repeated the diagnostic with nonnegative equal-weight family averages and their covariance-based variances as an exploratory sensitivity, rather than selecting a diagnostic by statistical significance.

Frontiers_Reproducibility_Materials_R1_1.zip contains the executed R scripts, input hashes, model-level datasets, results, source-linked amendment records and session information. Frontiers_Supplementary_Data_R2_1.xlsx supplies the extracted data and traceable classifications. Both files accompany this submission. Submitted comparator models are separated from the active revised analyses; copyrighted source reports are not redistributed.

# S11 Additional model details and source amendments

The active workbook contains all 26 revision models, coefficient-level CR2 inference, covariance and ICC assumptions, and complete leave-one-family-out results. The 137 additional candidates extend the submitted register to 316 records without adding reports or trial families. Descriptive-only and overlapping records must not be counted as pooled effects. A separate Scope_Dispositions sheet records 66 identified domain/configuration entries across nine reports, including all five teaching configurations, both message-framing strata and distinct questionnaire domains. These entries expand the descriptive inventory, not the fitted effect count. The version crosswalk documents every integrated numerical candidate.

The scope inventory distinguishes incompatible rank summaries, adjusted estimates without a compatible standardizer/covariance, unidentified treatment allocation, unresolved source-population/scoring contradictions and unavailable original category-specific evidence. In Carcelén-Fraile, some subscale cells are present but remain provisionally withheld because report-level contradictions are unresolved; this is not a claim that each subscale is intrinsically non-quantifiable. Folleto’s qualitative accounts of calmness are not substituted for its pictorial competence/acceptance questionnaires. For Sit, the abridged companion remains identified as a companion rather than the original report. These limitations are not coded as zero effects.

Core leave-one-family-out means ranged from 0.553 to 1.196, with only two families remaining in each model. These describe influence, not established robustness. In the broader exploratory sample-size analysis, 22 of 28 families had one consistent pair of analyzed arm sizes. The sample-size predictor coefficient was 3.085 (SE = 2.141; 95% CI [-1.382, 7.552]; p = .165). The interval included zero, so this diagnostic did not establish a size association. GLS assigned negative weights to 2 outcomes in 2 families. The nonnegative equal-weight sensitivity gave 2.937, 95% CI [-1.461, 7.334], p = .179. These results cannot exclude publication bias or distinguish selective reporting from differences in design, constructs and context.

Source amendments include Yan’s 591 pupils with valid questionnaires at both assessments out of 715 consenting pupils, contrasting with a total of 733 in the source Table 5 arm headers, Chan’s narrative SE and inconsistent table interval, Xu’s table/prose ICC discrepancy, and differing source labels for the MOGBA game-perception factors. Individual source calculations remain conditional on the listed reporting assumptions.

A complete report-ID inventory does not establish full participant-overlap adjudication or verification of every instrument. Completed source checks and unresolved fields remain distinguished in Outcome_Mapping, Source_Numeric_Cells and Scope_Dispositions. These targeted revision checks do not constitute a new exhaustive independent duplicate extraction of all 74 reports. No formal GRADE certainty assessment was performed.

# S12. Changes from the submitted synthesis

The comparison starts from the submitted 179-record dataset, not an intermediate revision checkpoint. Report inclusion remains 74 reports and 70 trial families. The 316-record revision register includes 137 additional candidates, some retained only for description; these are not 137 new pooled effects. The Supplementary Data sheet Submitted_Model_Changes lists each old/new model membership, record ID, reason and source. Version_Crosswalk covers all 316 candidates and preserves old and revised numerical values.

Core: all five retained IDs remain in the same three families. Xu_2025_02 (social interaction) and Xu_2025_03 (emotional experience) are components of the retained self-control total at the same endpoint. They were removed from the total-plus-components mean, not discarded from the evidence register. A separate sensitivity replaces the total with all four components. This changes 7 submitted effects to 5, not 3 families to fewer families.

Adjacent: nine of the 12 submitted IDs remain. Chan_AplusFMS_2019_teacher_support describes perceived teaching context, not student functioning. Yan_05_cluster_adjusted and Yan_06_cluster_adjusted measure introjected and external regulation, whose increase cannot automatically be labeled a favorable psychological change. They are retained separately. Four source-supported outcomes enter: R1_Chan_perceived_FMS, R1_MOGBA_game_perception, R1_MOGBA_game_perception_others and R1_Yan_autonomous_motivation. Thus, 12 − 3 + 4 = 13 effects, still from five families. Their source reporting discrepancies remain documented; inclusion is not a claim that these discrepancies are resolved.

The broader controlled synthesis changes from 71 effects/27 families to 126 effects/28 families: 55 submitted effect IDs remain, 16 leave this synthesis, and 71 enter. The additions comprise recoverable older unpooled records and newly extracted candidate outcomes, not 71 new reports. Removal reasons include overlapping components, context or nonuniform constructs, acute outcomes, and incompatible or unidentified analyzed samples. Added contrasts use the stated source-adjusted or raw-post external-ICC rules, with shared-control dependence retained within trial family. Table S12 and Submitted_Model_Changes separate membership changes from numerical corrections to retained IDs. No result was selected for its direction or significance.

**Table S12**. Submitted-to-revised controlled analysis membership

| Analysis | Old effects / families | Retained IDs | Removed IDs | Added IDs | Revised effects / families | Net effects |
| --- | --- | --- | --- | --- | --- | --- |
| core | 7 / 3 | 5 | 2 | 0 | 5 / 3 | -2 |
| adjacent | 12 / 5 | 9 | 3 | 4 | 13 / 5 | 1 |
| broader | 71 / 27 | 55 | 16 | 71 | 126 / 28 | 55 |
